# Inference of Genotype–Phenotype Relationships in the Antigenic Evolution of Human Influenza A (H3N2) Viruses

**DOI:** 10.1371/journal.pcbi.1002492

**Published:** 2012-04-19

**Authors:** Lars Steinbrück, Alice Carolyn McHardy

**Affiliations:** 1 Department for Algorithmic Bioinformatics, Heinrich Heine University, Düsseldorf, Germany; 2 Max-Planck Research Group for Computational Genomics and Epidemiology, Max-Planck Institute for Informatics, Saarbrücken, Germany; Imperial College London, United Kingdom

## Abstract

Distinguishing mutations that determine an organism's phenotype from (near-) neutral ‘hitchhikers’ is a fundamental challenge in genome research, and is relevant for numerous medical and biotechnological applications. For human influenza viruses, recognizing changes in the antigenic phenotype and a strains' capability to evade pre-existing host immunity is important for the production of efficient vaccines. We have developed a method for inferring ‘antigenic trees’ for the major viral surface protein hemagglutinin. In the antigenic tree, antigenic weights are assigned to all tree branches, which allows us to resolve the antigenic impact of the associated amino acid changes. Our technique predicted antigenic distances with comparable accuracy to antigenic cartography. Additionally, it identified both known and novel sites, and amino acid changes with antigenic impact in the evolution of influenza A (H3N2) viruses from 1968 to 2003. The technique can also be applied for inference of ‘phenotype trees’ and genotype–phenotype relationships from other types of pairwise phenotype distances.

## Author Summary

The molecular evolution of any organism is described by changes in the genotype resulting from genetic drift or selection to maintain or establish fitness under the given environmental conditions. Identification of phenotype-defining changes and their distinction from (near-) neutral (‘hitchhikers’) ones is a fundamental challenge in genome research. The standard approach involves time- and cost-intensive mutation experiments, which are typically low throughput, due to their experimental nature. We have developed a computational method for the inference of phenotypic impact of genotypic changes that is applicable to any system, within or across species, where homologous genetic sequences and associated pairwise phenotype distances are available. We demonstrate the accuracy of our method by application to the human influenza A (H3N2) virus. This exemplary system is of particular interest, as recognizing changes in the antigenic phenotype and a viral strains' capability to evade pre-existing host immunity is important for the production of efficient vaccines. We accurately identified known sites and amino acid changes with antigenic impact over 35 years of evolution, and provide further details on individual antigenically relevant changes in the evolution of influenza A (H3N2) viruses.

## Introduction

Influenza viruses are responsible for ∼500,000 deaths annually and are a substantial threat to human health [1]. Besides seasonal infections caused by human viruses, four major pandemics over the last 100 years have resulted in ∼50 million deaths worldwide [2]–[4]. The viruses are classified into three genera (A, B, C), all from the *Orthomyxoviridae* family, which comprises single-stranded, negative sense RNA viruses. Influenza A and B viruses evolve rapidly and continuously accumulate amino acid changes in the antibody-binding (epitope) sites of the surface proteins, resulting in changes in antigenicity. Thus, novel ‘antigenic types’ regularly appear and rise to predominance, causing worldwide epidemics despite existing vaccination programs [5], [6]. Influenza A viruses are further categorized into subtypes based on the composition of their surface proteins, hemagglutinin (H or HA) and neuraminidase (N or NA). In the human population, the subtypes H1N1 and H3N2 are currently circulating [7]. Both global population structure and geographic migration patterns are known to influence the evolution of H3N2. Russell *et al.* suggested East–Southeast Asia to serve as a global reservoir, from which seasonal epidemics in temperate zones are seeded [8]. Other regions, such as China or USA, might serve as seeding regions, too, and migration from and to other tropical regions than East-Southeast Asia is thought to have a significant influence on the global dynamics [9], [10].

To monitor genetic and antigenic changes, the World Health Organization (WHO) runs a global surveillance program [11]. Quantification of viral antigenic phenotypes is done with the hemagglutination inhibition (HI) assay, which measures the ability of an antiserum to inhibit the agglutination of red blood cells by a viral antigen [12]. Antigenic cartography, involving multidimensional scaling of log-normalized HI titers, subsequently generates an accurate low-dimensional representation of the antigenic distances between antigen–antiserum pairs [5], [13]. If a novel antigenic type with increasing prevalence is detected, the vaccine composition, consisting of two strains of influenza A (H3N2 and H1N1) and one strain of influenza B, is updated to include an antigenically closer match.

Antigenic cartography of influenza A (H3N2) isolates from 1968 to 2003 revealed that antigenic types circulate for 3.3 years, on average, in worldwide epidemics before being replaced by a successor [5]. A comparison of antigenic and genetic maps showed that, the antigenic impact of genetic changes varies, depending on the nature of the amino acids exchanged, their structural positioning and epistatic interactions with other sites. Subsequent studies have incorporated both antigenic and genetic data for predicting antigenically novel strains [14]–[16]. Additionally, many groups have investigated the influence of sequence positions and sequence variation on viral evolution, based on different computational criteria [17]–[24].

Even though the general principles governing the antigenic evolution of influenza A viruses are well studied, computational methods for directly determining the antigenic impact of individual amino acid exchanges do not yet exist. Such analyses currently require time- and cost-intensive experimental characterization of mutant viruses [5]. On the other end of the spectrum, antigenic cartography allows identification of ‘cluster difference substitutions’, comprising all near-conserved changes that distinguish consecutive antigenic clusters.

We describe a method for the inference of ‘antigenic trees’, which is based on a least-squares optimization (LSO) procedure of fitting pairwise antigenic distances onto an evolutionary tree for the major antigenic determinant of influenza A. It is a computational method allowing for a more fine-grained resolution of the antigenic impact of individual changes than antigenic cartography without time- and cost-intensive experiments. Application to HA sequences and serological data from human influenza A (H3N2) viral isolates from 1968 to 2003 determined the antigenic impact of all branch-associated amino acid changes for this time period. Our technique identified known antigenic types and the amino acid changes associated with the type transitions. For sufficiently resolved branches, the antigenic impact of individual exchanges could be quantified. The method furthermore found known and novel key HA sites and changes in antigenic evolution.

## Results

**Figure 1:**
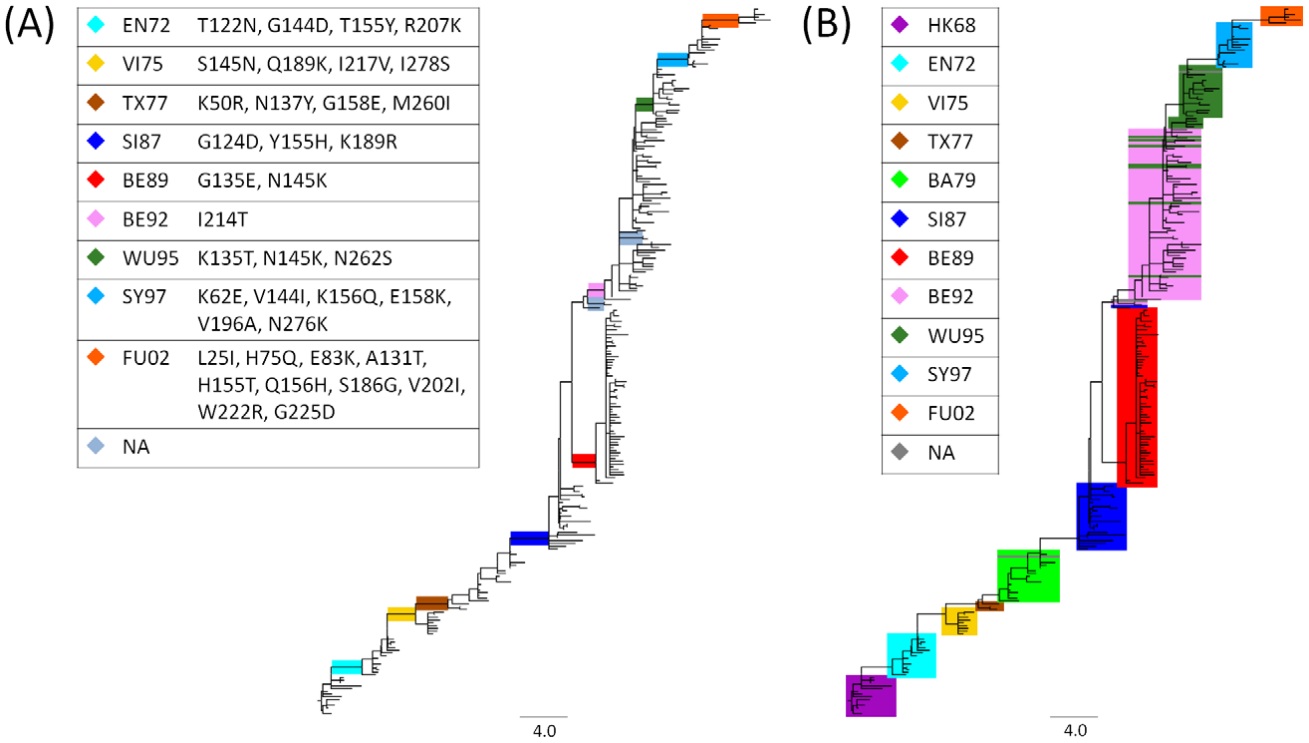
Antigenic tree for influenza A (H3N2) viruses. Branch lengths represent antigenic distances (maximum of up- and down-weights for each branch) inferred from a maximum likelihood tree of 258 hemagglutinin sequences of seasonal influenza A (H3N2) virus isolates and serological data. (A) Colored edges show antigenic type transitions, with internal branches with high average antigenic weights (≥1.0 antigenic units). Gray-blue edges represent high weight branches leading to a subtree with three isolates or less, representing low abundance types. (B) Isolates are color-coded by antigenic clusters according to Smith *et al.* (2004). Three isolates (A/Christchurch/4/85, A/Hong Kong/34/90 and A/Netherlands/172/96) are only present as antisera and were not assigned a cluster label.

We applied our method to infer an antigenic tree from genetic sequences of the hemagglutinin segment and serological data (HI titers of antigen-antiserum pairs) for 258 influenza A (H3N2) isolates sampled between 1968 and 2003 [5]. Antigenic branch lengths were determined by fitting the antigenic distances between viral isolates (the antigens) and antisera raised against reference strains to the branches of a maximum likelihood tree (see Materials & Methods). Antigenic branch lengths were realized as two independent weights (up and down) and represented the antigenic properties of antigens and antisera in the tree. The antigenic path length between two isolates, corresponding to the sum of the branch weights (either up- or down-weight, depending on the direction in the tree) for all connecting branches on the path between them in the tree, reflected their overall antigenic distance (**Figure 1**, high resolution **Figures S1 and S2**).

To investigate how accurately antigenic distances were fitted onto the tree, we evaluated its ability to predict unseen antigenic distances by leave-one-out cross validation [25]. In this experiment, an antigenic tree is inferred from all but one antigenic distance and then is applied to predict the left out distance. A predicted distance corresponds to the antigenic path length between the two respective isolates in the tree (see Materials & Methods). This was repeated for every antigenic distance and the overall accuracy of predicting antigenic distances estimated by the absolute prediction error and the root mean squared error (RSME) averaged over all leave-one-out experiments (see Materials & Methods). The leave-one-out absolute prediction error was 0.86 antigenic units (∼ a two-fold dilution, SD 0.72) and the correlation measured by Pearson's correlation coefficient between predicted and measured values was 0.86. Using placement on an antigenic map estimated from the same data, Smith *et al.* report an average absolute prediction error of 0.83 antigenic units (SD 0.67) and a Pearson's correlation coefficient of 0.80 for 481 measurements of antigenic distances [5]. The RMSE penalizes large prediction errors more than small prediction errors, and is a well suited measure of predictive accuracy. For our method, the leave-one-out RMSE is 1.12 antigenic units, corresponding to approximately a two-fold dilution. This is comparable to the ten-fold cross validation RMSE of Cai *et al.* on this data set (1.05 antigenic units) [26], who used a matrix completion algorithm prior to multi dimensional scaling. Our method therefore performs similarly to antigenic cartography in predicting antigenic distances, with a slightly larger error but also a slightly higher correlation between predicted and measured values. This is despite the fact that inferring antigenic branch lengths for an antigenic tree allows far fewer degrees of freedom than an antigenic map, where the data is not forced on a fixed structure. Note that for the prediction of antigenic distances, other well-suited methods also exist [26], [27].

As we infer a tree topology from nucleotide sequences, branches might be without any amino acid changes and thus lack explanatory power if they are assigned antigenic weights. This allows accommodating measurement errors in HI titers in antigenic branch weights or variation caused by changes in other viral antigens, such as the surface glycoprotein neuraminidase. HI titers are imprecise, as they reflect two-fold dilutions instead of quantitative estimates, and are often highly variable, with measurements varying between experiments and laboratories. For instance, the two isolates A/Finland/220/92 and A/Stockholm/20/91 have the same nucleotide sequence, and hence no changes on their respective tip branches (tips), but differ strongly in their HI values, where A/Finland/220/92 shows an antigenic distance from the same antisera that is, on average, ∼1.0 antigenic units (a two-fold dilution) larger than that of A/Stockholm/20/91. Note that, in general, even though neuraminidase may influence the HI titers, the WHO recommends application of the HI assay under conditions where its influence is negligible [28]. To incorporate a possible influence of neuraminidase activity one may use concatenated viral sequences (hemagglutinin and neuraminidase) and fit antigenic distances on a tree topology inferred from these sequences. If doing so, one should first ensure that reassortment events have not resulted in larger topological changes between the HA and NA genealogies during the analyzed time period [6], [29]. In case of larger topopological changes due to segment reassortment, a joint tree is inferred for data which cannot be described by a tree-like evolutionary history, overall, and the results are likely to be only partially informative.

On average, internal branches *without* amino acid changes have weights of 0.30 (up) and 0.21 (down), respectively. Less noise occurs on the tree trunk, which represents the viral lineage surviving over time, with 0.19 (up-weight) and 0.19 (down-weight) assigned, on average. Interestingly, the average antigenic weight of branches *with* amino acid changes is higher on the tree trunk than for all internal branches (up = 0.52, down = 0.61 vs. up = 0.44, down = 0.46). This is in agreement with an expected fitness advantage for viral isolates with larger antigenic changes, and therefore preferential fixation and establishment appear as changes on the tree trunk.

### Antigenic types resolved in the tree

Antigenic types are clearly distinguished by high average weights (≥1.0 antigenic units) in the antigenic tree (see Materials & Methods). Exclusion of branches leading to subtrees with three or less isolates, representing undersampled groups, identified nine branches defining type transitions (**Table 1**) and ten antigenic types. Abbreviations for these (HK68, EN72, VI75, TX77, SI87, BE89, BE92, WU95, SY97 and FU02) are used as in Smith *et al.* (2004) [5]. SY97, for instance, denotes antigenically similar A/Sydney/5/1997-like strains. The average antigenic distances of these branches range from 1.0 (SI87–BE89) to 2.6 antigenic units (WU95–SY97; **Table 1**, **Figure 1A**). Eight of the nine type transition branches are on the trunk of the tree, which represents the influenza A (H3N2) lineage surviving over time. An exception is BE89, which is located in a subtree that has become extinct.

**Table 1:** Internal branches with high average antigenic weights (≥1.0 antigenic units) and according antigenic types in comparison to antigenic clusters identified by antigenic cartography (branches leading to three or less isolates are excluded).

Type transition	Branch amino acid changes	Weights (up/down/avg)	Trunk	Additional amino acid changes	Weights (up/down/avg)	Trunk	Smith *et al.*
HK68–EN72	**T122N**, **G144D**, **T155Y**, **R207K**	2.6/0.4/1.5	x	L3F, **N188D**	0.9/0.2/0.5	x	3.4
EN72–VI75	**S145N**, **Q189K**, **I217V**, **I278S**	0.6/2.4/1.5	x	**N53D**, **N137S**, **L164Q**, **F174S**, **N193D**, **R201K**, **I213V**, **I230V**	0.0/1.0/0.5		4.4
VI75–TX77	**K50R**, **N137Y**, **G158E**, **M260I**	0.6/2.8/1.7	x	**E82K**	1.0/-/0.5		3.4
TX77–BA79				**N133S**, **P143S**, **G146S**, **K156E**, **T160K**, **Q197R**, **V217I**	1.4/0.0/0.7	x	3.3
				D2N, **N53D**, **N54S**, **I62K**, **D172G**, **V244L**	0.0/0.3/0.2	x	
BA79–SI87	**G124D**, **Y155H**, **K189R**	0.2/3.3/1.7	x				4.9
SI87–BE89	G135E, **N145K**	2.0/0.0/1.0					4.6
BE89–BE92	I214T	1.4/1.1/1.3	x	**E156K**, **E190D**, N193S, L226Q, **T262N**	1.0/0.0/0.5	x	7.8
				**S133D**	0.0/0.4/0.2	x	
BE92–WU95	K135T, **N145K**, N262S	1.5/1.1/1.3	x				4.6
WU95–SY97	**K62E**, V144I, **K156Q**, **E158K**, **V196A**, **N276K**	2.5/2.6/2.6	x				4.7
SY97–FU02	**L25I**, **R50G**, **H75Q**, **E83K**, **A131T**, **H155T**, **Q156H**, S186G, **V202I**, **W222R**, **G225D**	1.8/3.2/2.5	x				3.5

Branch amino acid changes indicate the corresponding branches, where changes in bold were also found by Smith *et al.* (2004), and weights give the respective up, down and average branch weights. Multiple branches that can be mapped to a single antigenic type are separated by dashed lines. Additional amino acid changes indicate branches that carry further mutations found to be cluster transition substitutions by Smith *et al.* (2004). For some branches, the down-weight was not defined, as no antiserum was in the respective subtree. Branches that can be mapped to multiple type transitions are shown at the first mapping only. Smith *et al.* (2004) present average distances between consecutive antigenic clusters, whereas average antigenic branch weights give a minimum distance between consecutive antigenic types. Note that on branches with multiple changes not all changes have to contribute to the antigenic weight, though their individual impacts could not be resolved with the dataset (unsampled viral isolates).

The setting of the threshold parameter for identification of antigenic types in the tree influences the performance of our method (**Table S5**). The selected threshold of 1.0 antigenic unit identified nine of ten antigenic type transitions found by antigenic cartography [5]. The TX77–BA79 transition was not predicted with our method in this setting, as the weights of the corresponding branch were slightly below the threshold (up-weight 1.4, down-weight 0.0). Our method resolves antigenically relevant changes between successive antigenic types in several cases to several successive branches. Therefore, a higher threshold of 2.0 antigenic units for individual branches (a four-fold dilution), as suggested to distinguish antigenically diverse viral strains [11], does not allow distinction between different antigenic groups (only if the transition is not well resolved in the data and the antigenic impact of multiple changes is summarized on a single branch). On the other hand, choosing a lower threshold of 0.5 antigenic units selects twelve additional type-defining branches (**Table S5**, **Figure S3**). Among these is the TX77-BA79 type-defining branch that corresponds to an antigenic cluster transition according to antigenic cartography [5]. Furthermore, four of these additional branches define antigenic subtypes that were distinct enough to warrant a vaccine update. A more detailed discussion of type-defining branches at the threshold of 0.5 antigenic units can be found in the supporting material (**Text S1**). Note, that the choice of the threshold distance is equivalent to find a minimal antigenic distance to distinguish groups of antigenically and genetically similar viral isolates. This is different from the question whether two specific viral isolates are antigenically similar or not, although both tasks are related to each other.

For the nine jointly identified type transitions, seven agree 100% in terms of the assigned viral isolates. For the BE89–BE92 transition, the isolate A/Netherlands/938/1992 is placed within BE92 using antigenic cartography and as preceding BE92 by our technique. Isolate assignment differs the most for the BE92–WU95 transition. This is likely to be caused by multiple occurrences of N145K, which is, according to Smith *et al.* (2004) [5], the change that defines the BE92–WU95 transition and has a major antigenic impact in that context (2.6 antigenic units). It was already noted by Smith *et al.* that isolates classified by antigenic cartography within WU95 are placed in the vicinity of BE92 in a tree. Our analysis agrees with these findings (**Figure 1B**). We found that for each branch adjacent to these disagreeing placements, N145K is present (isolates of the antigenic type WU95 located in the area of BE92), with large branch-associated antigenic weights (an average up-weight of 1.3), similar to the type-defining branch of WU95 (up-weight 1.5). This indicates that N145K has a large antigenic impact for all these isolates and, interestingly, was evolutionary volatile during that period.

Analysis of up- and down-weights for type-defining branches allows us to determine a direction for antigenic impact. For example, the branch separating HK68 and EN72 has a weight of 2.6 (up)/0.4 (down), which means that isolates of HK68 are antigenically more similar to sera raised against EN72 than vice versa. The opposite example represents the SY97–FU02 transition, where the corresponding branch weight is 1.8 (up)/3.2 (down), which means that SY97 isolates are more distant from antisera raised against FU02 than vice versa. Both examples are in agreement with results published by the WHO [30], [31].

As influenza A evolution in the analyzed data set is characterized by an underlying cluster structure, both antigenic types and antigenic clusters allow determination of cluster-difference or antigenic type associated substitutions. However, antigenic types (inferred by our method) and antigenic clusters (inferred by antigenic cartography) have different interpretations. Antigenic types represent sets of viral isolates showing similar evolutionary (defined by the phylogenetic tree) and antigenic (defined by the antigenic branch lengths) patterns. Antigenic cluster are solely defined by antigenic patterns and are determined by a k-means clustering approach. In datasets with less well-defined cluster structure, the k-means approach would hardly result in robust clusters and identification of phenotype-associated changes would be more difficult, whereas our method would likely be able to resolve phenotype-genotype relationships up to the level of resolution supported by the data.

### Substitutions in antigenic type transitions

Amino acid changes from eight of nine type transitions identified by both antigenic cartography and the antigenic tree include the cluster difference substitutions described in Smith *et al.* (2004) [5] (**Table 1**). Smith *et al.* define ‘cluster difference substitutions’ as changes in conserved residues between two consecutive antigenic clusters (conserved meaning present in at least *n*−1 isolates within a cluster of size *n*). For five transitions, all cluster difference substitutions are on the type-defining branch (BA79–SI87, SI87–BE89, BE92–WU95, WU95–SY97 and SY97–FU02). For three transitions (EN72–VI75, VI75–TX77 and HK68–EN72), the substitutions were resolved to several branches with different antigenic branch weights, which allows a more fine-grained distinction. The 12 substitutions of the EN72–VI75 transition were assigned to two consecutive branches, one with high and one with moderate antigenic weights. The branch with S145N, Q189K, I217V and I278S has a high antigenic weight, indicating that one or several of these have a very large antigenic impact. For the HK68–EN72 and the VI75–TX77 transitions, the substitutions were resolved to two consecutive branches with high and moderate antigenic weights, too.

For BE89–BE92, the amino acid changes differ from cluster difference substitutions. Here, the cluster difference substitutions are found on branches that precede the type-defining branch. The type-defining branch carries the change I214T, while the cluster difference substitutions map to two preceding branches with lower antigenic weights. I214T has not been mentioned in the literature before and is reversed downwards in the tree on a branch without any assigned antigenic weight. Thus, either the measurements here were too noisy to resolve the correct branch, or this position has an antigenic impact as an epistatic effect, allowing for the preceding changes to become antigenically effective. Support for a potential epistatic effect of this change can be found by detailed analysis of individual HI measurements for two isolates (A/Hong Kong/34/1990 and A/Netherlands/938/1992), which already have the preceding branch changes for BE92 but not the I214T change. On average, all antigens labeled BE92 by Smith *et al.* have a large antigenic distance (greater 4.7 antigenic units) from the antiserum A/Hong Kong/34/1990. A/Netherlands/938/1992 is similar to A/Hong Kong/34/1990, with an antigenic distance of 0.7 to this antiserum.

Four branches with type transitions (SI87–BE89, BE92–WU95, WU95–SY97 and SY97–FU02) include additional changes besides the cluster difference substitutions. For SI87–BE89, the change G135E is present, in addition to N145K. G135E appears twice more in the tree, with an average up-weight of 0.64. This indicates that it may also have an antigenic effect in SI87–BE89. For BE92–WU95, the changes K135T and N262S are present on the type-defining branch, in addition to N145K. Both are located in the antibody binding sites [32] and became fixed following their appearance on this trunk branch.

In a recent (unpublished) study, Koel *et al.* (*Koel et al.; Antigenic evolution of influenza A (H3N2) virus is dictated by 7 residues in the hemagglutinin protein; 2nd International Influenza Meeting, Münster; 2011*) determined by site-directed mutagenesis changes at seven positions in the HA protein (145, 155, 156, 158, 159, 189 and 193) responsible for significant phenotypic diversity in the evolution of influenza A (H3N2). We also find that for eight of the nine identified type-defining branches changes occur at five of these positions (no changes at positions 159 and 193 are involved in antigenic type transitions), which further confirm the relevance of these sites for antigenic evolution (**Table 1**). Note that, besides these five residues changes at 23 other positions map to the type-defining branches which not all have to contribute to the antigenic weight, though their individual impacts could not be further resolved with the dataset (unsampled viral isolates).

### Antigenic impact of individual amino acid changes and sites

We examined amino acid changes with strong antigenic relevance according to (i) the impact of all changes at a specific site and (ii) the impact of a specific change. In the first case, we determined all positions where at least three changes occurred, and the mean and median of the branch weights (up- or down-weight) were not less than one antigenic unit. Missing weights, e.g. where down-weights were not defined because no antiserum was raised for the corresponding subtree, were excluded from the calculations. Seven positions, 112, 137, 144, 155, 156, 189 and 208, satisfy these criteria (**Table S2 and S3**). All except position 112 are part of the antibody binding sites of HA1 [32]. Positions 137, 155 and 156 are also part of the receptor binding site [33]. Positions 155 and 189 may be particularly important, as all changes occur on the tree trunk and are part of type transitions. The importance of H155T and Q156H was also verified for the FU02 transition [34]. For positions 137, 144, and 156, several changes map to the tree trunk (three of six, four of nine, and one of three, respectively), indicating their antigenic relevance. Changes at position 112 explain single isolate variations, as all occur on tips. The antigenic impact of these changes may be due to hitchhiking effects, as they occur only in combination with other changes.

Next, we identified changes occurring at least three times in the tree with a mean and median antigenic weight (up- or down-weight) of more than one unit (**Table S4**). Again, missing weights were excluded from the calculations. Five changes satisfy these conditions. Four of these (K62E, N145K, L226Q and T248I) occur at positions in antibody binding sites [32]. N145K was experimentally verified to have a large antigenic impact [5]. K62E is part of the WU95–SY97 transition and has a high weight assigned on two further tips. Finally, of the eight occurrences of L226Q, seven appear between 1990 and 1996 for isolates of the BE92 type, indicative of a fitness effect for this antigenic type in particular. Interestingly, the reverse change, Q226L, is known to play a role in receptor binding specificity for the adaptation of bird viruses to the human host [35]–[38]. T248I had a high weight only in combination with other changes, indicating a potential epistatic effect. Besides these four changes, we identified V112I, which only appeared on tips and explains single isolate variations.

We searched for changes with moderate antigenic impact (more than 0.5 antigenic units) which identified seven further changes (**Table S2**). G135E is part of the SI87–BE89 transition (see above) and E156K was shown to impact immune escape in mice [39]. Both are located in the antibody binding sites [32]. For several additional changes, the importance was not immediately obvious, as they (i) occurred only in combination with other changes, (ii) exhibited a high weight only in combination with other changes (Q80K), (iii) only appeared on tips (S186I, S199P and V226I) or (iv) had high weights assigned only on tips and low weights on internal branches (A138T). In cases (i) and (ii), this may be the result of epistatic or hitchhiking effects, where epistasis may be more likely for (ii). Case (iii) changes are rare and explain single isolate sequence variations. This also seems to be likely in case (iv), where the effect on the tips is amplified due to other effects or amino acid changes. Notably, all case (iii) changes are also categorized as case (i) changes. Of all changes, E156K occurs once on the tree trunk. All changes appear at several points in time for different antigenic types, which indicates a potential antigenic influence. Furthermore, for five changes (G135E, A138T, E156K, S186I and V226I), the respective site was identified as being under positive selection [17].

In a recent (unpublished) study, Koel *et al.* (*Koel et al.; Antigenic evolution of influenza A (H3N2) virus is dictated by 7 residues in the hemagglutinin protein; 2nd International Influenza Meeting, Münster; 2011*) showed by site-directed mutagenesis that changes at seven positions in the HA protein (145, 155, 156, 158, 159, 189 and 193) are responsible for large antigenic changes, all except two are part of antigenic cluster transitions, over the 35 year time period. Of these, 155, 156 and 189 are also identified as generally important by our default method. If single isolate variations are excluded from the analysis, position 158 is also identified. For the other two positions (145 and 159) we identified changes with high antigenic weights (e.g. N145K and S159Y; **Table S2**). For position 193, evidence of antigenic importance could be found in our analysis if using ancestral character state reconstruction with maximum parsimony (see Supplement). Thus, our results also support the relevance of the sites proposed by Koel *et al.* (2011), even though they are not entirely comparable due to differences in experimental set up. Koel *et al.* analyzed prototype viruses with the amino acid consensus sequences of antigenic clusters and introduced only the specific changes between these prototype viruses, while our method also considers genetic and antigenic variations between other viral strains of the dataset.

## Discussion

The antigenic impact of amino acid substitutions in the antigenic evolution of influenza A viruses can reliably be determined by time- and cost-intensive experimental analysis. As an alternative, we present a computational technique for inferring the antigenic impact of amino acid changes. Our method determines antigenic branch lengths for a given tree topology by fitting pairwise antigenic distances between isolates onto the tree with LSO. For inference of the tree, any state-of-the-art method can be used. A comparison between maximum likelihood, maximum parsimony and neighbor-joining trees showed that all resulted in similar prediction errors (leave-one-out absolute prediction error: 0.86, 0.87 and 0.87 antigenic units, respectively; correlation between predicted and measured by Pearson's correlation coefficient was 0.86 for all three methods). The antigenic impact of the branch-associated amino acid changes is determined by reconstructing the branch-associated amino acid changes with maximum likelihood [40]; other techniques, such as maximum parsimony or Bayesian reconstruction, could also be used [41], [42]. A comparison between maximum likelihood and maximum parsimony ancestral character state reconstruction showed that these differed only in minor aspects, with the maximum likelihood reconstruction being an intermediate between accelerated and delayed transition in case of ties with maximum parsimony reconstruction. However, we did observe that more trunk branches were not assigned changes based on maximum likelihood reconstruction, which decreased the interpretability of antigenic weights in some cases.

We studied the antigenic evolution of the influenza A (H3N2) virus from 1968 to 2003 with antigenic trees inferred from data described in Smith *et al.* (2004) [5]. This allowed us to identify areas and branches in the tree corresponding to known antigenic types and transitions between these types. Analysis of antigenic weights identified seven sites in the HA1 domain of HA that were repeatedly associated with high antigenic impact. Additionally, our method identified five amino acid changes with high antigenic weights at several places in the antigenic tree. The sites and substitutions identified by our method may be of particular relevance for influenza A (H3N2) virus antigenic evolution, which has not been described before. For six of the seven positions found by site-directed mutagenesis to defining antigenic clusters for the 35 year time period (*Koel et al.; Antigenic evolution of influenza A (H3N2) virus is dictated by 7 residues in the hemagglutinin protein; 2nd International Influenza Meeting, Münster; 2011*), changes with high antigenic weights were identified with our technique, thus further supporting their relevance for influenza A (H3N2) evolution. The additional sites detected by our method could be more relevant for genetic and antigenic variations between viral strains in our data set not resulting in antigenic cluster transitions. These were not analyzed by Koel *et al.*, who characterized antigenic differences of prototype viruses with the amino acid consensus sequences of the antigenic clusters.

As the dataset covers 35 years of viral evolution with a relatively small number of isolates, not all substitutions could be resolved to individual branches and their individual antigenic impacts inferred. A denser sampling of data points would allow a more precise decoding of the genotype–antigenicity relationships, as viral isolates were unevenly sampled across the 35 years. The median number of viral isolates available per year between 1989 and 1997 was 15, whereas for the remaining years only three isolates per year were sampled (median). This unequal sampling is reflected in resolution of mutations to specific branches. Between 1989 and 1997, 19% of the branches with assigned changes carry three or more changes, whereas for the other years this is the case for 37% of the branches.

Our method allows inference of genotype to phenotype relationships from genetic sequences and associated pairwise phenotypic distances between individuals of a population or different taxa. We demonstrated the usefulness of this technique for analyzing the antigenic impact of amino acid changes in the evolution of human influenza A. An application of our method could be in influenza A virus surveillance. Here, it could be used to identify isolates and associated changes with large antigenic impact, which need to be identified for vaccine strain updates prior to an antigenic type transitions [43]. However, our method is not restricted to the analysis of influenza viruses or antigenic distance information but can be applied to the study of any system, be it within or across species, where homologous genetic sequences and associated pairwise phenotype distances are available. The software is available upon request from the authors.

## Materials and Methods

### Inferring the phenotypic impact of amino acid changes in protein evolution

Our idea is to adapt the least-squares optimization (LSO) technique of Cavalli-Sforza and Edwards [44] for phylogenetic inference to the problem of identifying the phenotypic impact of amino acid changes in protein evolution. The original method of Cavalli-Sforza and Edwards [44] identifies branch weights representing genetic distances according to the least-squares criterion for a tree topology. We applied this technique to infer ‘antigenic trees’, representing the antigenic evolution of the major surface protein of human influenza A virus (H3N2) over a 35-year period. In our adaptation, branch lengths represent antigenic distances inferred from HI assay data for human influenza A viruses and a maximum likelihood tree of the HA1 domain of hemagglutinin. Reconstruction of the amino acid changes associated with the branches of the tree allows us to infer the antigenic impact of the branch-associated amino acid changes. If sufficient data is available to resolve individual changes to individual branches, our method returns an estimate of the antigenic impact of the individual exchanges.

**Figure 2:**
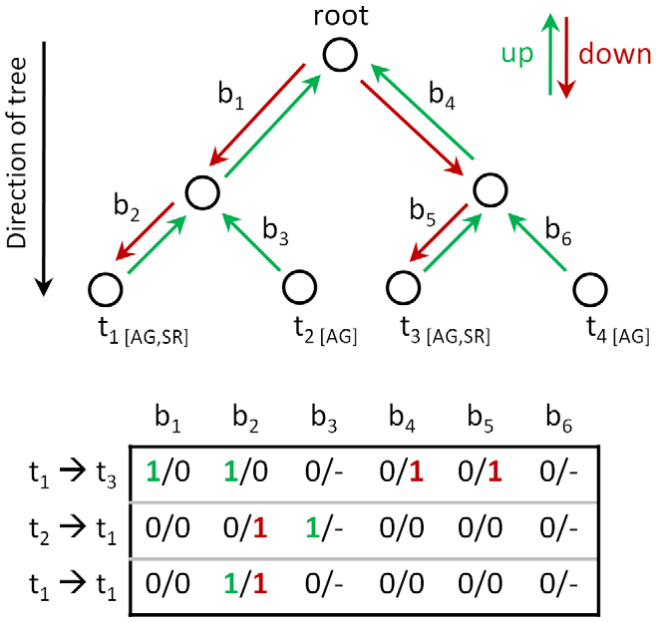
Schematic drawing demonstrating the up/down tree concept. For the two taxa *t_2_* and *t_4_*, no antiserum is present, and thus, *b_3_* and *b_6_* only have up-weights. A path from *t_1_* to *t_3_* would use the up-weights of branch *b_1_* and *b_2_*, and the down-weights of branch *b_4_* and b_5_. Similarly, the path from *t_2_* to *t_1_* would use the up-weight of branch *b_3_* and the down-weight of branch *b_2_*. Notably, the path from *t_1_* to *t_1_*, namely the antigenic distance from antigen *t_1_* to the antiserum raised against strain *t_1_*, would use the up-weight and the down-weight of branch *b_1_*.

In LSO, one minimizes the sum of squares between the given distances *D* and predicted distances *d:*

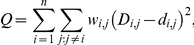
where *W* is the weight matrix for the different error terms, which were set to one here. The predicted distances *d_i,j_* are the sum of the branch weights on the path between leaf *i* and leaf *j*. Here, 
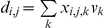
, where *x_i,j,k_* equals one if branch *k* is on the path between leaves *i* and *j* in the phylogenetic tree and zero otherwise. Thus, we search for the best setting for the branch weights *v_k_*. While evolutionary distances are usually used in this approach, here, we map antigenic distances to represent branch-specific weights. To restrict the branch weights to positive values, we used the Lawson–Hanson algorithm for non-negative LSO [45]. Because the antigenic distances here are asymmetric (i.e. *d_i,j_* 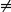 *d_j,i_*) and because the antigen and antiserum raised against the same viral strain do not necessarily have the same position in the antigenic space [13], we introduce the concept of up–down trees. In up–down trees, viral strains are mapped to the leaves representing the corresponding antigen as well as the antiserum, and every branch is assigned two independent weights, the up- and the down-weight. Every path between two taxa *i* and *j* in the tree can be separated into the set of branches from taxon *i* to the least common ancestor (LCA) of *i* and *j*, and the branches from taxon *j* to the LCA. Now, the path between antigen *i* and antiserum *j* involves only the up-weights on branches from taxon *i* to the LCA and only the down-weights on branches from taxon *j* to the LCA (**Figure 2**).

### Performance measures

To evaluate how accurately antigenic distances were fitted onto the tree, we used four performance measures in leave-one-out cross validation experiments: mean absolute error (MAE), root mean squared error (RMSE), standard deviation (SD) and Pearson's correlation coefficient (CC). In leave-one-out cross validation, an antigenic tree is inferred from all but one antigenic distances and then is applied to predict the left out distance. A predicted distance corresponds to the antigenic path length between the two respective isolates in the tree (see above). This was repeated for every antigenic distance. Given *n* observed distances *D_i,j_* and predicted distances *d_i,j_* the performance measures are defined as follows:
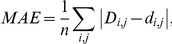


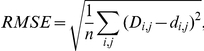


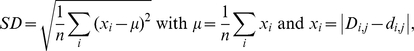


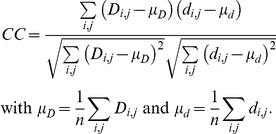



### Up-weights and down-weights in the tree

Antigenic branch lengths are realized as two independent weights, allowing for a detailed analysis of the underlying structure of the antigenic data. Up-weights represent the antigenic distance from isolates below this branch to every other isolate outside of this subtree, whereas down-weights represent distances from isolates outside of the subtree to the isolates below this branch. Thus, the branch weight types reveal different properties of the subtree. Let *e* be the branch going upwards from the least common ancestor of an antigenically homogenous group of viruses (a type) in the tree. The up-weight of *e* defines the degree to which the antigenic type is separated from other antigenic types according to antisera in other parts of the tree, i.e. how well antigens of this type are neutralized by antisera raised against other types. The down-weight of *e* defines the degree to which the antigenic type is separated from other types based on antisera *within* this part of the tree, i.e. how well other antigenic types are neutralized by antisera of this type. The antigenic weights of two types often differ, which is not surprising, as antigenic distances are not symmetric. For tip branches, the two weights define the different behavior of the antiserum and antigen of a viral strain. The up-weight reflects the antigenic properties of the isolate, whereas the down-weight reflects the antigenic weight of the antiserum raised against the viral isolate.

In case no antiserum is present in a subtree, down-weights are undefined and assignment of up-weights becomes ambiguous as they form linear combinations. To resolve this, optimization is done only on the up-weights leading to leaves in the according subtree. Afterwards, up-weights of the internal branches are set to the minimum of the up-weights on the branches leading to the respective child nodes (these up-weights are accordingly reduced by the minimum) in a bottom-up traversal. The rationale behind this is that if no additional information is present antigenic weights should rather be a common feature of a subgroup of taxa rather than single isolate variation for every taxon in the subgroup.

### Phylogenetic inference

Hemagglutinin (HA) sequences from 258 seasonal human influenza A (H3N2) virus isolates from 1968 to 2003 and that were used by Smith *et al.* (2004) [5] were downloaded from the Influenza Virus Resource (IVR) [46] (**Table S1**). Alignments of DNA and protein sequences, restricted to positions 1 to 363 (sites without missing data that appeared in more than 80% of the sequences), were created with Muscle [47] and manually curated. Trees were inferred with PhyML v3.0 [48] under the general time reversal GTR+I+Γ_4_ model, with the frequency of each substitution type, the proportion of invariant sites (I) and the Gamma distribution of among-site rate variation, with four rate categories (Γ_4_), estimated from the data. Subsequently, the tree topology and branch lengths of the maximum likelihood tree inferred with PhyML were optimized for 200,000 generations with Garli v0.96b8 [49]. Isolate A/duck/33/1980 was used as outgroup to root the tree and subsequently removed from the further analysis.

For placement of amino acid changes on the tree branches, protein sequences for the HA1 domain of HA (excluding the additional sites used for a higher resolution of the tree during the tree inference step) were assigned to the leaves of the tree inferred from nucleotide sequences. Ancestral character states were reconstructed under the maximum likelihood criterion using PAML v4.5 [50] under the JTT+Γ_4_+F model [51], with the frequency of each amino acid and the Gamma distribution of among-site rate variation, with four rate categories (Γ_4_), estimated from the data. Based on the reconstructed ancestral sequences for the internal nodes and leaf node sequences, amino acid changes were assigned to the individual tree branches.

### Antigenic data

HI assay data from Smith *et al.* (2004) was used and normalized according to these researchers' methods [5]. For each antigen *i*, antiserum *j* and the corresponding HI titer *h_i,j_*, the distance was set as *d_i,j_ = log_2_(max(h_j_)/h_i,j_)*, where *max(h_j_)* is the maximum entry for antiserum *j*. The dataset comprises 4,215 measured values between 273 antigens and 79 reference sera. As not all strains were available in the IVR, 18 antigens and 9 reference sera could not be mapped to a genetic sequence and were excluded from the analysis. Additionally, threshold values (e.g. <10, indicating the lower bound in the HI assay below which dilutions are not measured) were excluded from the analysis, as these values define only long-distance relationships and we did not want to introduce a potential bias by setting these entries to fixed values. In case of multiple antisera raised to the same viral strain, median values of the distances were used.

### Definition of antigenic types

Antigenic types in the antigenic tree can be distinguished by selecting type-defining branches according to a threshold distance. The threshold was set to 1.0 antigenic units for average weights (average of up- and down-weights), such that all branches are selected whose average weights are at least twice as high as the average weights of all internal branches. To exclude undersampled groups, all branches leading to subtrees with three or less isolates were excluded.

## Supporting Information

Figure S1 Antigenic tree with branch lengths representing antigenic distances (maximum of up- and down weights for each branch) inferred from a maximum likelihood tree of 258 hemagglutinin sequences of seasonal influenza A (H3N2) virus isolates and serological data. Isolates are color-coded by antigenic clusters according to Smith *et al.* (2004). Three isolates (A/Christchurch/4/85, A/Hong Kong/34/90 and A/Netherlands/172/96) are only present as antiserum and were not assigned a cluster label. Changes on terminal branches are colored in black, whereas changes on internal branches are colored in blue.
(PDF)

Figure S2 Antigenic tree with branch lengths representing antigenic distances (maximum of up- and down weights for each branch) inferred from a maximum likelihood tree of 258 hemagglutinin sequences of seasonal influenza A (H3N2) virus isolates and serological data. Isolates are color-coded by antigenic clusters according to Smith *et al.* (2004). Three isolates (A/Christchurch/4/85, A/Hong Kong/34/90 and A/Netherlands/172/96) are only present as antiserum and were not assigned a cluster label. Branch labels depict assigned weights (up/down).
(PDF)

Figure S3 Antigenic tree for influenza A (H3N2) viruses. Branch lengths represent antigenic distances (maximum of up- and down-weights for each branch) inferred from a maximum likelihood tree of 258 hemagglutinin sequences of seasonal influenza A (H3N2) virus isolates and serological data. Colored edges show antigenic type transitions, with internal branches with high average antigenic weights (≥1.0 antigenic units, coloring according to **Figure 1A**) or moderate antigenic weights ≥0.5 antigenic units (coloring as gradient from the higher order antigenic type). Subscript _2_ indicates that a branch was a direct successor of the according type-defining branch (except of branch (i), who is a predecessor of the according type-defining branch). Subscript _sub_ indicates a subdivision of an antigenic type without a direct matching of a reference strain.
(TIF)

Table S1 GenBank accession numbers of the used hemagglutinin sequences.
(DOC)

Table S2 Summary of changes in the phylogenetic tree. Branch amino acid changes refer to the set of changes mapped to a specific branch. For some branches, the down-weight was not defined, as no antiserum was in the respective subtree.
(DOC)

Table S3 Positions with multiple changes in the phylogenetic tree and high antigenic weights (mean and median ≥1 antigenic unit, highlighted in bold). ‘Tip’ indicates leaf branches.
(DOC)

Table S4 Changes with multiple occurrences in the phylogenetic tree and high antigenic weights (mean and median ≥1 antigenic unit). ‘Tip’ indicates leaf branches. Down-weights are omitted, as all changes were identified using up-weights.
(DOC)

Table S5 Type-defining branches selected by different thresholds for average branch weights. Branches (1)–(9) were selected as type-defining branches at a threshold distance of 1.0 antigenic units. Branches (i)–(xii) reveal further subdivision of antigenic types at a threshold distance of 0.5 antigenic units. Asterisks mark branches whose sibling branch leads to a single isolate. Subscript _2_ indicates that a branch is a direct successor of a type-defining branch (except for branch (i), which is a predecessor of the type-defining branch). Subscript _sub_ indicates a subdivision of an antigenic type without a directly known reference strain.
(DOC)

Text S1 Influence of threshold distance on type-defining branches.
(DOC)
