# Supplementary figures and images for: Inference of Genotype–Phenotype Relationships in the Antigenic Evolution of Human Influenza A (H3N2) Viruses
Source: PLoS Comput Biol. 2012 Apr 19;8(4):e1002492. doi: 10.1371/journal.pcbi.1002492 (PMC3330098; doi:10.1371/journal.pcbi.1002492)

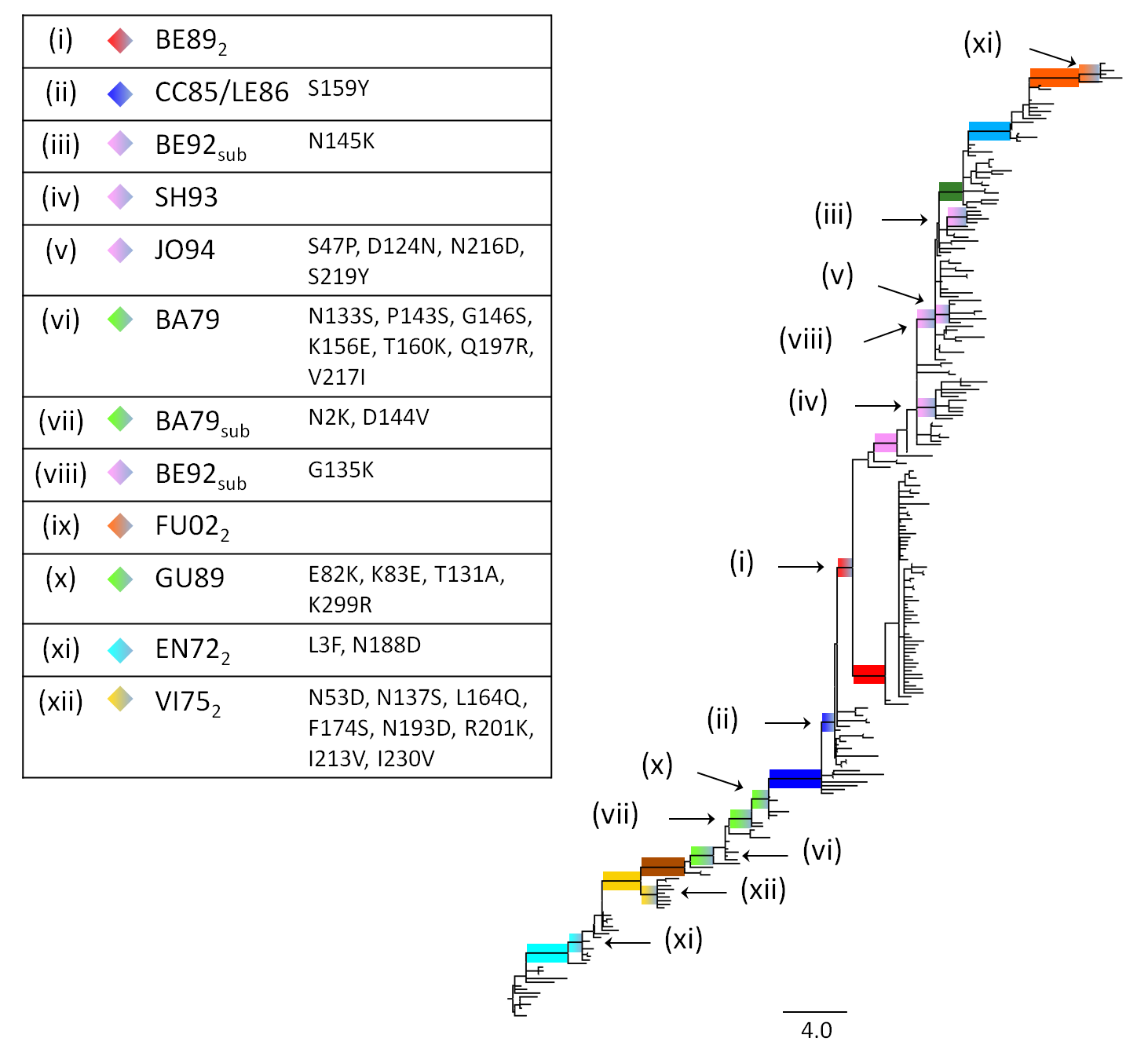

Supplement: Figure S3 — Antigenic tree for influenza A (H3N2) viruses. Branch lengths represent antigenic distances (maximum of up- and down-weights for each branch) inferred from a maximum likelihood tree of 258 hemagglutinin sequences of seasonal influenza A (H3N2) virus isolates and serological data. Colored edges show antigenic type transitions, with internal branches with high average antigenic weights (≥1.0 antigenic units, coloring according to Figure 1A ) or moderate antigenic weights ≥0.5 antigenic units (coloring as gradient from the higher order antigenic type). Subscript 2 indicates that a branch was a direct successor of the according type-defining branch (except of branch (i), who is a predecessor of the according type-defining branch). Subscript sub indicates a subdivision of an antigenic type without a direct matching of a reference strain. (TIF) [file pcbi.1002492.s003.tif]
