# Supplementary material for: Inference of Genotype–Phenotype Relationships in the Antigenic Evolution of Human Influenza A (H3N2) Viruses
Source: PLoS Comput Biol. 2012 Apr 19;8(4):e1002492. doi: 10.1371/journal.pcbi.1002492 (PMC3330098; doi:10.1371/journal.pcbi.1002492)
Supplement: Table S1 — GenBank accession numbers of the used hemagglutinin sequences. (DOC) [file pcbi.1002492.s004.doc]

AAA18781, AAA43145, AAB63692, AAB63706, AAB66725, AAB66732, AAB66764, AAB66778, AAB66792, AAB69809, AAB69826, AAB69828, AAB69830, AAB69838, AAB69845, AAB69847, AAC78096, AAC78098, AAF06949, AAF13705, AAF16422, AAF16454, AAF16495, AAK54149, AAK54151, AAT64666, AAT64667, AAT64668, AAT64669, AAT64670, AAT64671, AAT64672, AAT64673, AAT64674, AAT64675, AAT64676, AAT64677, AAT64678, AAT64679, AAT64680, AAT64681, AAT64682, AAT64683, AAT64684, AAT64685, AAT64686, AAT64687, AAT64688, AAT64689, AAT64690, AAT64691, AAT64692, AAT64693, AAT64694, AAT64695, AAT64696, AAT64697, AAT64698, AAT64699, AAT64700, AAT64701, AAT64702, AAT64703, AAT64704, AAT64705, AAT64706, AAT64707, AAT64708, AAT64709, AAT64710, AAT64711, AAT64712, AAT64713, AAT64714, AAT64715, AAT64716, AAT64717, AAT64718, AAT64719, AAT64720, AAT64721, AAT64722, AAT64723, AAT64724, AAT64725, AAT64726, AAT64727, AAT64728, AAT64729, AAT64730, AAT64731, AAT64732, AAT64733, AAT64734, AAT64735, AAT64736, AAT64737, AAT64738, AAT64739, AAT64740, AAT64741, AAT64742, AAT64743, AAT64744, AAT64745, AAT64746, AAT64747, AAT64748, AAT64749, AAT64750, AAT64751, AAT64752, AAT64753, AAT64754, AAT64755, AAT64756, AAT64757, AAT64758, AAT64759, AAT64760, AAT64761, AAT64762, AAT64763, AAT64764, AAT64765, AAT64766, AAT64767, AAT64768, AAT64769, AAT64770, AAT64771, AAT64772, AAT64773, AAT64774, AAT64775, AAT64776, AAT64777, AAT64778, AAT64779, AAT64780, AAT64781, AAT64782, AAT64783, AAT64784, AAT64785, AAT64786, AAT64787, AAT64788, AAT64789, AAT64790, AAT64791, AAT64792, AAT64793, AAT64794, AAT64795, AAT64796, AAT64797, AAT64798, AAT64799, AAT64800, AAT64801, AAT64802, AAT64803, AAT64804, AAT64805, AAT64806, AAT64807, AAT64808, AAT64809, AAT64810, AAT64811, AAT64812, AAT64813, AAT64814, AAT64815, AAT64816, AAT64817, AAT64818, AAT64819, AAT64820, AAT64821, AAT64822, AAT64823, AAT64824, AAT64825, AAT64826, AAT64827, AAT64828, AAT64829, AAT64830, AAT64831, AAT64832, AAT64833, AAT64834, AAT64835, AAT64836, AAT64837, AAT64838, AAT64839, AAT64840, AAT64841, AAT64842, AAT64843, AAT64844, AAT64845, AAT64846, AAT64847, AAT64848, AAT64849, AAT64850, AAT64851, AAT64852, AAT64853, AAT64854, AAT64855, AAT64856, AAT64857, AAT64858, AAT64859, AAT64860, AAT64861, AAT64862, AAT64863, AAT64864, AAT64865, AAT64866, AAT64867, AAT64868, AAT64869, AAT64870, AAT64871, AAT64872, AAT64873, AAT64876, AAT64877, AAT64878, AAT64879, AAT64880, AAT64881, AAT64882, AAT64883, AAT64884, AAT64885, AAT64886, ABP51971, ABQ10136, ABQ58926, ABQ58927, ABQ58928, ABQ58929, ABQ58930, ABQ58932, ABQ58940, BAA21070, CAA86540, CAA86541, CAA86543, CAA86548, CAA86549
