# Supplementary material for: Inference of Genotype–Phenotype Relationships in the Antigenic Evolution of Human Influenza A (H3N2) Viruses
Source: PLoS Comput Biol. 2012 Apr 19;8(4):e1002492. doi: 10.1371/journal.pcbi.1002492 (PMC3330098; doi:10.1371/journal.pcbi.1002492)
Supplement: Table S2 — Summary of changes in the phylogenetic tree. Branch amino acid changes refer to the set of changes mapped to a specific branch. For some branches, the down-weight was not defined, as no antiserum was in the respective subtree. (DOC) [file pcbi.1002492.s005.doc]

| Change | Branch amino acid changes | Weights (up/down) | Trunk | Tip |
| --- | --- | --- | --- | --- |
| D2N |  |  |  |  |
|  | D2N, N53D, N54S, I62K, D172G, V244L | 0.0 / 0.35 | x |  |
|  | D2N, S145I, S159N, T167S, P273S | 0.94 / - |  | x |
| N2K |  |  |  |  |
|  | N2K, D144V | 1.43 / 0.0 | x |  |
| L3F |  |  |  |  |
|  | L3F, N188D | 0.9 / 0.19 | x |  |
| F3L |  |  |  |  |
|  | F3L, T83K, T126N | 0.0 / 0.0 | x |  |
| L3I |  |  |  |  |
|  | L3I | 0.39 / 0.0 |  |  |
|  | L3I, A138S | 0.63 / 0.0 |  | x |
| G5V |  |  |  |  |
|  | G5V, Q33H, K92T | 0.0 / 0.0 |  |  |
| G5R |  |  |  |  |
|  | G5R, I62M, F79L, S145R, S159N, E190D, I213V, S219P, R229G, V244L | 0.49 / - |  | x |
| N6S |  |  |  |  |
|  | N6S, S47T | 0.06 / - |  | x |
| D7E |  |  |  |  |
|  | D7E | 2.59 / - |  | x |
| N8K |  |  |  |  |
|  | N8K, V244I | 0.74 / - |  |  |
| T10K |  |  |  |  |
|  | T10K, I34T, G129E, Q132E, G144D, S159R, N188D, A198T | 0.0 / 1.64 |  | x |
| P21S |  |  |  |  |
|  | P21S, H183L | 0.43 / 0.57 |  | x |
| L25I |  |  |  |  |
|  | L25I, R50G, H75Q, E83K, A131T, H155T, Q156H, S186G, V202I, W222R, G225D | 1.77 / 3.16 | x |  |
| I29L |  |  |  |  |
|  | I29L, F79L, Q226L | 0.67 / - |  | x |
| D31N |  |  |  |  |
|  | D31N, S145I | 0.0 / 0.74 | x |  |
|  | D31N | 0.33 / - |  |  |
| D31V |  |  |  |  |
|  | D31V, K50R, N54S, I62K, Q80K, N133D, C139F, R145K, G146R, S199P | 0.47 / 0.0 |  | x |
| Q33H |  |  |  |  |
|  | G5V, Q33H, K92T | 0.0 / 0.0 |  |  |
| H33N |  |  |  |  |
|  | H33N, A131D, D271N | 0.78 / 0.49 |  | x |
| I34V |  |  |  |  |
|  | I34V | 0.0 / - |  | x |
| I34T |  |  |  |  |
|  | T10K, I34T, G129E, Q132E, G144D, S159R, N188D, A198T | 0.0 / 1.64 |  | x |
| Q44H |  |  |  |  |
|  | Q44H, V144I, Y159H, S186I | 2.66 / 0.3 |  | x |
| S45R |  |  |  |  |
|  | S45R | 0.27 / - |  | x |
| S45N |  |  |  |  |
|  | S45N, V112I, N133D, T135A, L151W | - / 0.18 |  | x |
|  | S45N, F79L, A304P | 0.48 / - |  | x |
|  | S45N, G78D | 0.05 / - |  |  |
|  | S45N | 0.3 / - |  |  |
| S45G |  |  |  |  |
|  | S45G, N122K, A138T | 0.74 / 0.0 |  | x |
| S47P |  |  |  |  |
|  | S47P, D124N, N216D, S219Y | 0.62 / 0.9 |  |  |
| S47T |  |  |  |  |
|  | N6S, S47T | 0.06 / - |  | x |
| G49S |  |  |  |  |
|  | G49S, K158R, V204I, Y233H, N312K | 0.0 / - |  | x |
| K50R |  |  |  |  |
|  | K50R, N137Y, G158E, M260I | 0.64 / 2.76 | x |  |
|  | D31V, K50R, N54S, I62K, Q80K, N133D, C139F, R145K, G146R, S199P | 0.47 / 0.0 |  | x |
| R50G |  |  |  |  |
|  | L25I, R50G, H75Q, E83K, A131T, H155T, Q156H, S186G, V202I, W222R, G225D | 1.77 / 3.16 | x |  |
|  | R50G, S199P, S247C | 0.77 / 0.0 |  | x |
| R50K |  |  |  |  |
|  | R50K | 1.53 / - |  | x |
|  | R50K | 0.0 / - |  |  |
|  | R50K, T121I, V196I | 0.0 / - |  |  |
| R50I |  |  |  |  |
|  | R50I, K207R | 0.91 / 0.38 |  | x |
| N53D |  |  |  |  |
|  | D2N, N53D, N54S, I62K, D172G, V244L | 0.0 / 0.35 | x |  |
|  | N53D, N137S, L164Q, F174S, N193D, R201K, I213V, I230V | 0.0 / 1.05 |  |  |
| D53N |  |  |  |  |
|  | D53N | 0.0 / - |  |  |
|  | D53N, K326R | 0.0 / - |  | x |
| D53G |  |  |  |  |
|  | D53G | 1.58 / 0.0 |  | x |
|  | D53G | 1.05 / - |  | x |
| N54S |  |  |  |  |
|  | D2N, N53D, N54S, I62K, D172G, V244L | 0.0 / 0.35 | x |  |
|  | D31V, K50R, N54S, I62K, Q80K, N133D, C139F, R145K, G146R, S199P | 0.47 / 0.0 |  | x |
| S54I |  |  |  |  |
|  | S54I, N144I, S199P | 1.39 / - |  | x |
| S54G |  |  |  |  |
|  | S54G, L59I, A198S, G275D | 0.49 / - |  | x |
| P55H |  |  |  |  |
|  | P55H | 0.26 / - |  | x |
| R57Q |  |  |  |  |
|  | R57Q | 0.0 / 0.69 | x |  |
|  | R57Q | 0.11 / 0.97 |  |  |
| I58V |  |  |  |  |
|  | I58V, S115P, N216Y, T235A | 0.51 / - |  | x |
|  | I58V, T262I | 0.76 / - |  | x |
| L59I |  |  |  |  |
|  | S54G, L59I, A198S, G275D | 0.49 / - |  | x |
| I62K |  |  |  |  |
|  | D2N, N53D, N54S, I62K, D172G, V244L | 0.0 / 0.35 | x |  |
|  | I62K, K176R | 0.79 / - |  | x |
|  | D31V, K50R, N54S, I62K, Q80K, N133D, C139F, R145K, G146R, S199P | 0.47 / 0.0 |  | x |
| K62E |  |  |  |  |
|  | K62E, V144I, K156Q, E158K, V196A, N276K | 2.51 / 2.62 | x |  |
|  | K62E, N246D | 0.63 / - |  | x |
|  | K62E | 1.42 / - |  | x |
| K62R |  |  |  |  |
|  | K62R, E158D, T192I, A198S, E280G | 0.0 / - |  | x |
| I62M |  |  |  |  |
|  | G5R, I62M, F79L, S145R, S159N, E190D, I213V, S219P, R229G, V244L | 0.49 / - |  | x |
| I62V |  |  |  |  |
|  | I62V, L226Q | 0.0 / - |  | x |
| D63N |  |  |  |  |
|  | D63N, S193N | 0.46 / 0.31 | x |  |
|  | D63N | 0.09 / - |  |  |
| I67M |  |  |  |  |
|  | I67M | 0.73 / - |  |  |
| I67V |  |  |  |  |
|  | I67V, G124S | 0.0 / 0.0 |  |  |
| H75Q |  |  |  |  |
|  | L25I, R50G, H75Q, E83K, A131T, H155T, Q156H, S186G, V202I, W222R, G225D | 1.77 / 3.16 | x |  |
| H75N |  |  |  |  |
|  | H75N, I121T | 0.0 / 0.0 |  |  |
| V78G |  |  |  |  |
|  | V78G | 0.0 / 0.0 | x |  |
| G78D |  |  |  |  |
|  | G78D | 0.4 / - |  |  |
|  | G78D | 0.11 / - |  |  |
|  | S45N, G78D | 0.05 / - |  |  |
| F79L |  |  |  |  |
|  | S45N, F79L, A304P | 0.48 / - |  | x |
|  | I29L, F79L, Q226L | 0.67 / - |  | x |
|  | F79L, I121T, N122K | 0.3 / - |  |  |
|  | G5R, I62M, F79L, S145R, S159N, E190D, I213V, S219P, R229G, V244L | 0.49 / - |  | x |
| Q80K |  |  |  |  |
|  | Q80K | 0.8 / - |  |  |
|  | Q80K, T128A, V226I, N246S, M268I | 1.28 / - |  | x |
|  | D31V, K50R, N54S, I62K, Q80K, N133D, C139F, R145K, G146R, S199P | 0.47 / 0.0 |  | x |
| D81N |  |  |  |  |
|  | D81N | - / 0.09 |  |  |
| E82K |  |  |  |  |
|  | E82K, K83E, T131A, K299R | 0.81 / 0.29 | x |  |
|  | E82K | 1.0 / - |  |  |
| T83K |  |  |  |  |
|  | F3L, T83K, T126N | 0.0 / 0.0 | x |  |
| K83E |  |  |  |  |
|  | E82K, K83E, T131A, K299R | 0.81 / 0.29 | x |  |
| E83K |  |  |  |  |
|  | L25I, R50G, H75Q, E83K, A131T, H155T, Q156H, S186G, V202I, W222R, G225D | 1.77 / 3.16 | x |  |
| V88I |  |  |  |  |
|  | V88I | 0.04 / 0.81 |  | x |
|  | V88I, D188E | 0.0 / 0.0 |  |  |
| K92T |  |  |  |  |
|  | G5V, Q33H, K92T | 0.0 / 0.0 |  |  |
|  | K92T | 0.0 / 0.34 |  |  |
| K92E |  |  |  |  |
|  | K92E, L226Q, R299K | 1.17 / 1.81 |  | x |
| K92N |  |  |  |  |
|  | K92N | 0.93 / - |  | x |
| K92R |  |  |  |  |
|  | K92R | 0.07 / 0.0 |  | x |
| F94Y |  |  |  |  |
|  | F94Y | 0.0 / 0.0 | x |  |
|  | F94Y, E156K, S279Y | 0.74 / 0.51 |  | x |
| N96D |  |  |  |  |
|  | N96D, A198T, I226V, R229G | 0.4 / 0.96 |  | x |
| N96S |  |  |  |  |
|  | N96S | 0.15 / - |  | x |
|  | N96S | 0.72 / 0.0 |  |  |
|  | N96S, R201K, K259Q, D291N | 0.0 / - |  | x |
| D101E |  |  |  |  |
|  | D101E | 0.26 / - |  | x |
| P103Q |  |  |  |  |
|  | P103Q, T313N | 0.0 / - |  | x |
| P103S |  |  |  |  |
|  | P103S | 0.44 / - |  | x |
| A106V |  |  |  |  |
|  | A106V, S186G | 0.0 / 0.0 |  |  |
|  | A106V, G135R | 0.0 / - |  |  |
| V112I |  |  |  |  |
|  | V112I, N121T, N312K | 1.46 / 0.0 |  | x |
|  | S45N, V112I, N133D, T135A, L151W | - / 0.18 |  | x |
|  | V112I | 0.82 / 0.73 |  | x |
|  | V112I | 1.13 / - |  | x |
| S114A |  |  |  |  |
|  | S114A | 0.55 / - |  | x |
| S115P |  |  |  |  |
|  | I58V, S115P, N216Y, T235A | 0.51 / - |  | x |
| I121T |  |  |  |  |
|  | I121T, D124G, G172D, R197Q | 0.13 / 0.0 | x |  |
|  | I121T, E158D, R201K | 0.45 / 0.73 |  | x |
|  | H75N, I121T | 0.0 / 0.0 |  |  |
|  | I121T, E158D | 0.33 / 0.0 |  |  |
|  | F79L, I121T, N122K | 0.3 / - |  |  |
|  | I121T | 0.0 / - |  | x |
|  | I121T, P169S, I278V | 0.81 / - |  | x |
| T121N |  |  |  |  |
|  | T121N | 0.0 / 0.0 | x |  |
| N121T |  |  |  |  |
|  | V112I, N121T, N312K | 1.46 / 0.0 |  | x |
| T121I |  |  |  |  |
|  | R50K, T121I, V196I | 0.0 / - |  |  |
| T122N |  |  |  |  |
|  | T122N, G144D, T155Y, R207K | 2.6 / 0.41 | x |  |
| N122K |  |  |  |  |
|  | S45G, N122K, A138T | 0.74 / 0.0 |  | x |
|  | N122K | 0.0 / 0.05 |  |  |
|  | F79L, I121T, N122K | 0.3 / - |  |  |
| G124D |  |  |  |  |
|  | G124D, Y155H, K189R | 0.18 / 3.3 | x |  |
|  | G124D, G142E, D175E, K310R | 0.0 / 0.0 |  | x |
| D124G |  |  |  |  |
|  | I121T, D124G, G172D, R197Q | 0.13 / 0.0 | x |  |
| G124S |  |  |  |  |
|  | G124S, D133N | 0.0 / 0.33 | x |  |
|  | I67V, G124S | 0.0 / 0.0 |  |  |
| D124A |  |  |  |  |
|  | D124A | 0.81 / - |  | x |
| D124N |  |  |  |  |
|  | S47P, D124N, N216D, S219Y | 0.62 / 0.9 |  |  |
| D124E |  |  |  |  |
|  | D124E, K173R, R261H, E325D | 0.77 / 0.02 |  | x |
| T126N |  |  |  |  |
|  | F3L, T83K, T126N | 0.0 / 0.0 | x |  |
| T128A |  |  |  |  |
|  | T128A, A304P | 0.2 / - |  | x |
|  | Q80K, T128A, V226I, N246S, M268I | 1.28 / - |  | x |
| G129E |  |  |  |  |
|  | T10K, I34T, G129E, Q132E, G144D, S159R, N188D, A198T | 0.0 / 1.64 |  | x |
| T131A |  |  |  |  |
|  | E82K, K83E, T131A, K299R | 0.81 / 0.29 | x |  |
| A131T |  |  |  |  |
|  | L25I, R50G, H75Q, E83K, A131T, H155T, Q156H, S186G, V202I, W222R, G225D | 1.77 / 3.16 | x |  |
| A131D |  |  |  |  |
|  | H33N, A131D, D271N | 0.78 / 0.49 |  | x |
| Q132E |  |  |  |  |
|  | T10K, I34T, G129E, Q132E, G144D, S159R, N188D, A198T | 0.0 / 1.64 |  | x |
| N133S |  |  |  |  |
|  | N133S, P143S, G146S, K156E, T160K, Q197R, V217I | 1.44 / 0.0 | x |  |
| S133D |  |  |  |  |
|  | S133D | 0.0 / 0.37 | x |  |
| D133N |  |  |  |  |
|  | G124S, D133N | 0.0 / 0.33 | x |  |
|  | D133N | 0.0 / - |  | x |
| N133D |  |  |  |  |
|  | S45N, V112I, N133D, T135A, L151W | - / 0.18 |  | x |
|  | D31V, K50R, N54S, I62K, Q80K, N133D, C139F, R145K, G146R, S199P | 0.47 / 0.0 |  | x |
| S133N |  |  |  |  |
|  | S133N | 0.0 / - |  |  |
|  | S133N, S186I, V213R, V309G | 0.54 / - |  | x |
| G135K |  |  |  |  |
|  | G135K | 0.24 / 1.14 | x |  |
| K135T |  |  |  |  |
|  | K135T, N145K, N262S | 1.52 / 1.1 | x |  |
| T135A |  |  |  |  |
|  | S45N, V112I, N133D, T135A, L151W | - / 0.18 |  | x |
| K135E |  |  |  |  |
|  | K135E, N145K, N285D | 1.62 / 1.55 |  | x |
| G135E |  |  |  |  |
|  | G135E | 0.88 / 0.0 |  |  |
|  | G135E, N145K | 2.04 / 0.0 |  |  |
|  | G135E | 0.41 / - |  | x |
| E135K |  |  |  |  |
|  | E135K | 0.06 / - |  |  |
| G135R |  |  |  |  |
|  | A106V, G135R | 0.0 / - |  |  |
| E135D |  |  |  |  |
|  | E135D | 0.0 / - |  |  |
| N137Y |  |  |  |  |
|  | K50R, N137Y, G158E, M260I | 0.64 / 2.76 | x |  |
| Y137S |  |  |  |  |
|  | Y137S | 0.0 / 0.0 | x |  |
|  | Y137S | 0.3 / - |  | x |
| N137S |  |  |  |  |
|  | N53D, N137S, L164Q, F174S, N193D, R201K, I213V, I230V | 0.0 / 1.05 |  |  |
|  | N137S, G275D | 0.35 / - |  | x |
| S137G |  |  |  |  |
|  | S137G, K140R, I267M | 0.0 / - |  | x |
| A138T |  |  |  |  |
|  | S45G, N122K, A138T | 0.74 / 0.0 |  | x |
|  | A138T | 0.52 / - |  |  |
|  | A138T | 0.0 / - |  | x |
|  | A138T, D188E | 0.46 / - |  | x |
|  | A138T | 0.0 / - |  | x |
|  | A138T | 1.29 / 0.71 |  | x |
|  | A138T, D144N, V182I, V196I | 0.26 / 1.29 |  | x |
| A138S |  |  |  |  |
|  | A138S, T167A | 0.03 / - |  | x |
|  | L3I, A138S | 0.63 / 0.0 |  | x |
| C139F |  |  |  |  |
|  | D31V, K50R, N54S, I62K, Q80K, N133D, C139F, R145K, G146R, S199P | 0.47 / 0.0 |  | x |
| K140R |  |  |  |  |
|  | S137G, K140R, I267M | 0.0 / - |  | x |
| G142R |  |  |  |  |
|  | G142R | 0.0 / 0.0 | x |  |
|  | G142R, S186I, Q197H | 0.88 / - |  | x |
| R142S |  |  |  |  |
|  | R142S, L194I, V226I | 0.0 / 1.24 |  | x |
| G142E |  |  |  |  |
|  | G124D, G142E, D175E, K310R | 0.0 / 0.0 |  | x |
| P143S |  |  |  |  |
|  | N133S, P143S, G146S, K156E, T160K, Q197R, V217I | 1.44 / 0.0 | x |  |
| P143T |  |  |  |  |
|  | P143T | 0.31 / - |  |  |
| G144D |  |  |  |  |
|  | T122N, G144D, T155Y, R207K | 2.6 / 0.41 | x |  |
|  | T10K, I34T, G129E, Q132E, G144D, S159R, N188D, A198T | 0.0 / 1.64 |  | x |
| D144V |  |  |  |  |
|  | N2K, D144V | 1.43 / 0.0 | x |  |
| V144I |  |  |  |  |
|  | K62E, V144I, K156Q, E158K, V196A, N276K | 2.51 / 2.62 | x |  |
|  | Q44H, V144I, Y159H, S186I | 2.66 / 0.3 |  | x |
| I144N |  |  |  |  |
|  | I144N, D172E, T192I | 0.0 / 0.39 | x |  |
| N144D |  |  |  |  |
|  | N144D | 0.0 / - |  |  |
| N144I |  |  |  |  |
|  | S54I, N144I, S199P | 1.39 / - |  | x |
| D144N |  |  |  |  |
|  | A138T, D144N, V182I, V196I | 0.26 / 1.29 |  | x |
| S145I |  |  |  |  |
|  | D31N, S145I | 0.0 / 0.74 | x |  |
|  | S145I | 0.25 / - |  |  |
|  | D2N, S145I, S159N, T167S, P273S | 0.94 / - |  | x |
| I145S |  |  |  |  |
|  | I145S, V242I, D275G | 0.0 / 0.0 | x |  |
| S145N |  |  |  |  |
|  | S145N, Q189K, I217V, I278S | 0.57 / 2.42 | x |  |
|  | S145N | 0.73 / - |  | x |
| N145K |  |  |  |  |
|  | K135T, N145K, N262S | 1.52 / 1.1 | x |  |
|  | N145K | 1.25 / 0.27 |  |  |
|  | N145K | 0.89 / - |  | x |
|  | K135E, N145K, N285D | 1.62 / 1.55 |  | x |
|  | N145K | 1.63 / - |  |  |
|  | N145K, R208K, L226Q | 1.73 / 0.0 |  | x |
|  | N145K | 0.68 / - |  | x |
|  | G135E, N145K | 2.04 / 0.0 |  |  |
|  | N145K, R261L | 0.86 / - |  | x |
| K145N |  |  |  |  |
|  | K145N, K160R, V226I, T248I | 1.48 / 0.69 |  | x |
| S145R |  |  |  |  |
|  | G5R, I62M, F79L, S145R, S159N, E190D, I213V, S219P, R229G, V244L | 0.49 / - |  | x |
|  | S145R | 0.0 / 0.0 |  |  |
| R145K |  |  |  |  |
|  | D31V, K50R, N54S, I62K, Q80K, N133D, C139F, R145K, G146R, S199P | 0.47 / 0.0 |  | x |
| G146S |  |  |  |  |
|  | N133S, P143S, G146S, K156E, T160K, Q197R, V217I | 1.44 / 0.0 | x |  |
| S146G |  |  |  |  |
|  | S146G, Y159F | 0.67 / - |  | x |
| G146R |  |  |  |  |
|  | D31V, K50R, N54S, I62K, Q80K, N133D, C139F, R145K, G146R, S199P | 0.47 / 0.0 |  | x |
| F148L |  |  |  |  |
|  | F148L, N193K | 0.36 / - |  | x |
| L151W |  |  |  |  |
|  | S45N, V112I, N133D, T135A, L151W | - / 0.18 |  | x |
| T155Y |  |  |  |  |
|  | T122N, G144D, T155Y, R207K | 2.6 / 0.41 | x |  |
| Y155H |  |  |  |  |
|  | G124D, Y155H, K189R | 0.18 / 3.3 | x |  |
| H155T |  |  |  |  |
|  | L25I, R50G, H75Q, E83K, A131T, H155T, Q156H, S186G, V202I, W222R, G225D | 1.77 / 3.16 | x |  |
| K156E |  |  |  |  |
|  | N133S, P143S, G146S, K156E, T160K, Q197R, V217I | 1.44 / 0.0 | x |  |
|  | K156E, E158K | - / 0.0 |  | x |
| E156K |  |  |  |  |
|  | E156K, E190D, N193S, L226Q, T262N | 0.97 / 0.0 | x |  |
|  | E156K | 1.46 / - |  |  |
|  | E156K, I186V | 0.07 / 0.0 |  |  |
|  | F94Y, E156K, S279Y | 0.74 / 0.51 |  | x |
| K156Q |  |  |  |  |
|  | K62E, V144I, K156Q, E158K, V196A, N276K | 2.51 / 2.62 | x |  |
| Q156H |  |  |  |  |
|  | L25I, R50G, H75Q, E83K, A131T, H155T, Q156H, S186G, V202I, W222R, G225D | 1.77 / 3.16 | x |  |
| E156G |  |  |  |  |
|  | E156G, N246S | - / 0.0 |  | x |
| S157L |  |  |  |  |
|  | S157L | 0.01 / 0.64 | x |  |
|  | S157L, S209N | 0.47 / 0.0 |  | x |
| S157P |  |  |  |  |
|  | S157P | 0.67 / - |  | x |
| G158E |  |  |  |  |
|  | K50R, N137Y, G158E, M260I | 0.64 / 2.76 | x |  |
| E158K |  |  |  |  |
|  | K62E, V144I, K156Q, E158K, V196A, N276K | 2.51 / 2.62 | x |  |
|  | K156E, E158K | - / 0.0 |  | x |
| K158R |  |  |  |  |
|  | G49S, K158R, V204I, Y233H, N312K | 0.0 / - |  | x |
| E158D |  |  |  |  |
|  | K62R, E158D, T192I, A198S, E280G | 0.0 / - |  | x |
|  | I121T, E158D, R201K | 0.45 / 0.73 |  | x |
|  | I121T, E158D | 0.33 / 0.0 |  |  |
| S159Y |  |  |  |  |
|  | S159Y | 1.06 / 0.67 | x |  |
| Y159F |  |  |  |  |
|  | S146G, Y159F | 0.67 / - |  | x |
| Y159H |  |  |  |  |
|  | Q44H, V144I, Y159H, S186I | 2.66 / 0.3 |  | x |
| S159N |  |  |  |  |
|  | D2N, S145I, S159N, T167S, P273S | 0.94 / - |  | x |
|  | G5R, I62M, F79L, S145R, S159N, E190D, I213V, S219P, R229G, V244L | 0.49 / - |  | x |
| S159R |  |  |  |  |
|  | T10K, I34T, G129E, Q132E, G144D, S159R, N188D, A198T | 0.0 / 1.64 |  | x |
| T160K |  |  |  |  |
|  | N133S, P143S, G146S, K156E, T160K, Q197R, V217I | 1.44 / 0.0 | x |  |
| K160R |  |  |  |  |
|  | K145N, K160R, V226I, T248I | 1.48 / 0.69 |  | x |
| T160A |  |  |  |  |
|  | T160A | 0.0 / 1.09 |  | x |
| V163A |  |  |  |  |
|  | V163A | 0.02 / 0.0 | x |  |
| A163E |  |  |  |  |
|  | A163E | 0.55 / - |  | x |
| L164Q |  |  |  |  |
|  | N53D, N137S, L164Q, F174S, N193D, R201K, I213V, I230V | 0.0 / 1.05 |  |  |
| N165T |  |  |  |  |
|  | N165T | 0.0 / - |  | x |
| T167A |  |  |  |  |
|  | A138S, T167A | 0.03 / - |  | x |
|  | T167A | 0.87 / - |  | x |
| T167S |  |  |  |  |
|  | D2N, S145I, S159N, T167S, P273S | 0.94 / - |  | x |
| P169S |  |  |  |  |
|  | I121T, P169S, I278V | 0.81 / - |  | x |
| N171K |  |  |  |  |
|  | N171K, L226M, K264R, K292R | 0.66 / - |  | x |
| D172G |  |  |  |  |
|  | D2N, N53D, N54S, I62K, D172G, V244L | 0.0 / 0.35 | x |  |
| G172D |  |  |  |  |
|  | I121T, D124G, G172D, R197Q | 0.13 / 0.0 | x |  |
|  | G172D, V196I | 0.0 / - |  |  |
| D172E |  |  |  |  |
|  | I144N, D172E, T192I | 0.0 / 0.39 | x |  |
| N173K |  |  |  |  |
|  | N173K, I213V, N248T, K307R | 0.23 / 0.0 | x |  |
| K173R |  |  |  |  |
|  | D124E, K173R, R261H, E325D | 0.77 / 0.02 |  | x |
| F174V |  |  |  |  |
|  | F174V | 0.68 / 1.4 |  |  |
| F174S |  |  |  |  |
|  | N53D, N137S, L164Q, F174S, N193D, R201K, I213V, I230V | 0.0 / 1.05 |  |  |
| D175E |  |  |  |  |
|  | G124D, G142E, D175E, K310R | 0.0 / 0.0 |  | x |
| K176R |  |  |  |  |
|  | I62K, K176R | 0.79 / - |  | x |
| V182I |  |  |  |  |
|  | A138T, D144N, V182I, V196I | 0.26 / 1.29 |  | x |
| H183L |  |  |  |  |
|  | P21S, H183L | 0.43 / 0.57 |  | x |
| S186G |  |  |  |  |
|  | L25I, R50G, H75Q, E83K, A131T, H155T, Q156H, S186G, V202I, W222R, G225D | 1.77 / 3.16 | x |  |
|  | A106V, S186G | 0.0 / 0.0 |  |  |
| S186I |  |  |  |  |
|  | G142R, S186I, Q197H | 0.88 / - |  | x |
|  | Q44H, V144I, Y159H, S186I | 2.66 / 0.3 |  | x |
|  | S186I | 0.7 / 0.65 |  |  |
|  | S133N, S186I, V213R, V309G | 0.54 / - |  | x |
| I186V |  |  |  |  |
|  | E156K, I186V | 0.07 / 0.0 |  |  |
| N188D |  |  |  |  |
|  | L3F, N188D | 0.9 / 0.19 | x |  |
|  | T10K, I34T, G129E, Q132E, G144D, S159R, N188D, A198T | 0.0 / 1.64 |  | x |
| D188N |  |  |  |  |
|  | D188N | 0.05 / - |  | x |
| D188E |  |  |  |  |
|  | D188E, R220K | 0.54 / - |  |  |
|  | A138T, D188E | 0.46 / - |  | x |
|  | V88I, D188E | 0.0 / 0.0 |  |  |
| D188Y |  |  |  |  |
|  | D188Y, N193K, S278I | - / 0.18 |  | x |
| Q189K |  |  |  |  |
|  | S145N, Q189K, I217V, I278S | 0.57 / 2.42 | x |  |
| K189R |  |  |  |  |
|  | G124D, Y155H, K189R | 0.18 / 3.3 | x |  |
| R189S |  |  |  |  |
|  | R189S, Q226L | 0.62 / 0.13 | x |  |
| E190D |  |  |  |  |
|  | E156K, E190D, N193S, L226Q, T262N | 0.97 / 0.0 | x |  |
|  | E190D | 0.43 / - |  | x |
|  | G5R, I62M, F79L, S145R, S159N, E190D, I213V, S219P, R229G, V244L | 0.49 / - |  | x |
| D190V |  |  |  |  |
|  | D190V | 0.63 / 0.37 |  | x |
| T192I |  |  |  |  |
|  | I144N, D172E, T192I | 0.0 / 0.39 | x |  |
|  | T192I, V226I | 0.0 / - |  | x |
|  | K62R, E158D, T192I, A198S, E280G | 0.0 / - |  | x |
| S193N |  |  |  |  |
|  | D63N, S193N | 0.46 / 0.31 | x |  |
| N193S |  |  |  |  |
|  | E156K, E190D, N193S, L226Q, T262N | 0.97 / 0.0 | x |  |
|  | N193S | 0.0 / 0.86 |  |  |
| N193K |  |  |  |  |
|  | F148L, N193K | 0.36 / - |  | x |
|  | N193K | 0.21 / 0.32 |  | x |
|  | D188Y, N193K, S278I | - / 0.18 |  | x |
| N193D |  |  |  |  |
|  | N53D, N137S, L164Q, F174S, N193D, R201K, I213V, I230V | 0.0 / 1.05 |  |  |
| L194I |  |  |  |  |
|  | R142S, L194I, V226I | 0.0 / 1.24 |  | x |
|  | L194I | 0.0 / 1.09 |  | x |
| V196A |  |  |  |  |
|  | K62E, V144I, K156Q, E158K, V196A, N276K | 2.51 / 2.62 | x |  |
| V196I |  |  |  |  |
|  | V196I | 0.74 / - |  | x |
|  | G172D, V196I | 0.0 / - |  |  |
|  | R50K, T121I, V196I | 0.0 / - |  |  |
|  | A138T, D144N, V182I, V196I | 0.26 / 1.29 |  | x |
| I196V |  |  |  |  |
|  | I196V | 0.0 / - |  | x |
| Q197R |  |  |  |  |
|  | N133S, P143S, G146S, K156E, T160K, Q197R, V217I | 1.44 / 0.0 | x |  |
| R197Q |  |  |  |  |
|  | I121T, D124G, G172D, R197Q | 0.13 / 0.0 | x |  |
| Q197H |  |  |  |  |
|  | G142R, S186I, Q197H | 0.88 / - |  | x |
| A198S |  |  |  |  |
|  | S54G, L59I, A198S, G275D | 0.49 / - |  | x |
|  | K62R, E158D, T192I, A198S, E280G | 0.0 / - |  | x |
| A198T |  |  |  |  |
|  | N96D, A198T, I226V, R229G | 0.4 / 0.96 |  | x |
|  | T10K, I34T, G129E, Q132E, G144D, S159R, N188D, A198T | 0.0 / 1.64 |  | x |
| S199P |  |  |  |  |
|  | R50G, S199P, S247C | 0.77 / 0.0 |  | x |
|  | S54I, N144I, S199P | 1.39 / - |  | x |
|  | S199P, R229I, G275S | 0.0 / - |  | x |
|  | D31V, K50R, N54S, I62K, Q80K, N133D, C139F, R145K, G146R, S199P | 0.47 / 0.0 |  | x |
| R201K |  |  |  |  |
|  | I121T, E158D, R201K | 0.45 / 0.73 |  | x |
|  | N96S, R201K, K259Q, D291N | 0.0 / - |  | x |
|  | R201K | 1.54 / - |  | x |
|  | N53D, N137S, L164Q, F174S, N193D, R201K, I213V, I230V | 0.0 / 1.05 |  |  |
| R201G |  |  |  |  |
|  | R201G | 0.27 / - |  |  |
| V202I |  |  |  |  |
|  | L25I, R50G, H75Q, E83K, A131T, H155T, Q156H, S186G, V202I, W222R, G225D | 1.77 / 3.16 | x |  |
| T203I |  |  |  |  |
|  | T203I, N262T | 0.4 / - |  | x |
| V204I |  |  |  |  |
|  | G49S, K158R, V204I, Y233H, N312K | 0.0 / - |  | x |
| S205A |  |  |  |  |
|  | S205A, L226M, R229G | 1.17 / - |  | x |
| R207K |  |  |  |  |
|  | T122N, G144D, T155Y, R207K | 2.6 / 0.41 | x |  |
|  | R207K, L226P, I236T, T276A | 0.0 / - |  | x |
| K207R |  |  |  |  |
|  | K207R | 0.0 / - |  |  |
|  | R50I, K207R | 0.91 / 0.38 |  | x |
| R208K |  |  |  |  |
|  | N145K, R208K, L226Q | 1.73 / 0.0 |  | x |
| R208I |  |  |  |  |
|  | R208I | 2.0 / - |  |  |
| R208G |  |  |  |  |
|  | R208G | 0.0 / 1.19 |  | x |
| S209G |  |  |  |  |
|  | S209G, R229G | 0.0 / 0.19 |  | x |
| S209N |  |  |  |  |
|  | S209N, I213V, T262N | 0.0 / - |  |  |
|  | S157L, S209N | 0.47 / 0.0 |  | x |
| I213V |  |  |  |  |
|  | N173K, I213V, N248T, K307R | 0.23 / 0.0 | x |  |
|  | S209N, I213V, T262N | 0.0 / - |  |  |
|  | N53D, N137S, L164Q, F174S, N193D, R201K, I213V, I230V | 0.0 / 1.05 |  |  |
|  | G5R, I62M, F79L, S145R, S159N, E190D, I213V, S219P, R229G, V244L | 0.49 / - |  | x |
| V213R |  |  |  |  |
|  | S133N, S186I, V213R, V309G | 0.54 / - |  | x |
| I214T |  |  |  |  |
|  | I214T | 1.44 / 1.1 | x |  |
|  | I214T | 0.0 / 0.0 |  | x |
| T214I |  |  |  |  |
|  | T214I | 0.0 / 0.0 | x |  |
| N216Y |  |  |  |  |
|  | I58V, S115P, N216Y, T235A | 0.51 / - |  | x |
| N216D |  |  |  |  |
|  | S47P, D124N, N216D, S219Y | 0.62 / 0.9 |  |  |
| I217V |  |  |  |  |
|  | S145N, Q189K, I217V, I278S | 0.57 / 2.42 | x |  |
| V217I |  |  |  |  |
|  | N133S, P143S, G146S, K156E, T160K, Q197R, V217I | 1.44 / 0.0 | x |  |
| S219F |  |  |  |  |
|  | S219F | 0.33 / 0.07 |  |  |
|  | S219F | 0.18 / - |  | x |
| S219Y |  |  |  |  |
|  | S47P, D124N, N216D, S219Y | 0.62 / 0.9 |  |  |
|  | S219Y, P289S | 0.0 / - |  |  |
| S219P |  |  |  |  |
|  | S219P | 0.0 / 0.0 |  |  |
|  | S219P | 0.69 / 0.05 |  | x |
|  | G5R, I62M, F79L, S145R, S159N, E190D, I213V, S219P, R229G, V244L | 0.49 / - |  | x |
| R220K |  |  |  |  |
|  | D188E, R220K | 0.54 / - |  |  |
| W222R |  |  |  |  |
|  | L25I, R50G, H75Q, E83K, A131T, H155T, Q156H, S186G, V202I, W222R, G225D | 1.77 / 3.16 | x |  |
| G225D |  |  |  |  |
|  | L25I, R50G, H75Q, E83K, A131T, H155T, Q156H, S186G, V202I, W222R, G225D | 1.77 / 3.16 | x |  |
| L226Q |  |  |  |  |
|  | E156K, E190D, N193S, L226Q, T262N | 0.97 / 0.0 | x |  |
|  | L226Q, S231G | 2.0 / - |  | x |
|  | L226Q | 2.32 / 0.93 |  | x |
|  | N145K, R208K, L226Q | 1.73 / 0.0 |  | x |
|  | K92E, L226Q, R299K | 1.17 / 1.81 |  | x |
|  | L226Q | 0.71 / - |  | x |
|  | L226Q | 0.36 / 0.0 |  |  |
|  | I62V, L226Q | 0.0 / - |  | x |
| Q226L |  |  |  |  |
|  | R189S, Q226L | 0.62 / 0.13 | x |  |
|  | I29L, F79L, Q226L | 0.67 / - |  | x |
| L226I |  |  |  |  |
|  | L226I, S278N | 0.1 / 0.0 | x |  |
| I226V |  |  |  |  |
|  | I226V | 0.0 / 0.0 | x |  |
|  | N96D, A198T, I226V, R229G | 0.4 / 0.96 |  | x |
| V226I |  |  |  |  |
|  | K145N, K160R, V226I, T248I | 1.48 / 0.69 |  | x |
|  | R142S, L194I, V226I | 0.0 / 1.24 |  | x |
|  | Q80K, T128A, V226I, N246S, M268I | 1.28 / - |  | x |
|  | T192I, V226I | 0.0 / - |  | x |
| L226M |  |  |  |  |
|  | S205A, L226M, R229G | 1.17 / - |  | x |
|  | N171K, L226M, K264R, K292R | 0.66 / - |  | x |
| L226P |  |  |  |  |
|  | R207K, L226P, I236T, T276A | 0.0 / - |  | x |
| S227P |  |  |  |  |
|  | S227P | 1.51 / - |  |  |
| R229G |  |  |  |  |
|  | S209G, R229G | 0.0 / 0.19 |  | x |
|  | N96D, A198T, I226V, R229G | 0.4 / 0.96 |  | x |
|  | S205A, L226M, R229G | 1.17 / - |  | x |
|  | R229G, T248I | 1.55 / - |  | x |
|  | G5R, I62M, F79L, S145R, S159N, E190D, I213V, S219P, R229G, V244L | 0.49 / - |  | x |
| R229I |  |  |  |  |
|  | S199P, R229I, G275S | 0.0 / - |  | x |
| I230V |  |  |  |  |
|  | N53D, N137S, L164Q, F174S, N193D, R201K, I213V, I230V | 0.0 / 1.05 |  |  |
| S231G |  |  |  |  |
|  | L226Q, S231G | 2.0 / - |  | x |
| Y233H |  |  |  |  |
|  | G49S, K158R, V204I, Y233H, N312K | 0.0 / - |  | x |
|  | Y233H | 0.07 / 0.0 |  |  |
| T235A |  |  |  |  |
|  | I58V, S115P, N216Y, T235A | 0.51 / - |  | x |
| I236T |  |  |  |  |
|  | R207K, L226P, I236T, T276A | 0.0 / - |  | x |
| V242I |  |  |  |  |
|  | I145S, V242I, D275G | 0.0 / 0.0 | x |  |
| V244L |  |  |  |  |
|  | D2N, N53D, N54S, I62K, D172G, V244L | 0.0 / 0.35 | x |  |
|  | G5R, I62M, F79L, S145R, S159N, E190D, I213V, S219P, R229G, V244L | 0.49 / - |  | x |
| V244I |  |  |  |  |
|  | N8K, V244I | 0.74 / - |  |  |
| N246S |  |  |  |  |
|  | Q80K, T128A, V226I, N246S, M268I | 1.28 / - |  | x |
|  | E156G, N246S | - / 0.0 |  | x |
| N246D |  |  |  |  |
|  | K62E, N246D | 0.63 / - |  | x |
| S247C |  |  |  |  |
|  | R50G, S199P, S247C | 0.77 / 0.0 |  | x |
| S247R |  |  |  |  |
|  | S247R | 2.89 / 0.92 |  | x |
| N248T |  |  |  |  |
|  | N173K, I213V, N248T, K307R | 0.23 / 0.0 | x |  |
| T248I |  |  |  |  |
|  | K145N, K160R, V226I, T248I | 1.48 / 0.69 |  | x |
|  | R229G, T248I | 1.55 / - |  | x |
|  | T248I | 0.0 / - |  | x |
| K259Q |  |  |  |  |
|  | N96S, R201K, K259Q, D291N | 0.0 / - |  | x |
| M260I |  |  |  |  |
|  | K50R, N137Y, G158E, M260I | 0.64 / 2.76 | x |  |
| I260L |  |  |  |  |
|  | I260L | 1.57 / - |  |  |
| R261Q |  |  |  |  |
|  | R261Q | 1.59 / 0.42 |  | x |
| R261H |  |  |  |  |
|  | D124E, K173R, R261H, E325D | 0.77 / 0.02 |  | x |
| R261L |  |  |  |  |
|  | N145K, R261L | 0.86 / - |  | x |
| T262N |  |  |  |  |
|  | E156K, E190D, N193S, L226Q, T262N | 0.97 / 0.0 | x |  |
|  | S209N, I213V, T262N | 0.0 / - |  |  |
|  | T262N | 0.0 / - |  | x |
| N262S |  |  |  |  |
|  | K135T, N145K, N262S | 1.52 / 1.1 | x |  |
| N262T |  |  |  |  |
|  | T203I, N262T | 0.4 / - |  | x |
| T262I |  |  |  |  |
|  | I58V, T262I | 0.76 / - |  | x |
| K264R |  |  |  |  |
|  | N171K, L226M, K264R, K292R | 0.66 / - |  | x |
| I267V |  |  |  |  |
|  | I267V | 1.93 / - |  | x |
| I267M |  |  |  |  |
|  | S137G, K140R, I267M | 0.0 / - |  | x |
| M268I |  |  |  |  |
|  | Q80K, T128A, V226I, N246S, M268I | 1.28 / - |  | x |
|  | M268I | 0.0 / - |  | x |
| R269K |  |  |  |  |
|  | R269K | 1.49 / - |  | x |
| D271N |  |  |  |  |
|  | H33N, A131D, D271N | 0.78 / 0.49 |  | x |
| A272V |  |  |  |  |
|  | A272V | 0.84 / - |  |  |
| P273S |  |  |  |  |
|  | P273S | 0.41 / 0.62 |  |  |
|  | D2N, S145I, S159N, T167S, P273S | 0.94 / - |  | x |
| P273T |  |  |  |  |
|  | P273T | 0.29 / - |  | x |
| D275G |  |  |  |  |
|  | I145S, V242I, D275G | 0.0 / 0.0 | x |  |
| G275S |  |  |  |  |
|  | S199P, R229I, G275S | 0.0 / - |  | x |
| G275D |  |  |  |  |
|  | S54G, L59I, A198S, G275D | 0.49 / - |  | x |
|  | G275D | 0.0 / 0.49 |  |  |
|  | G275D | 0.0 / 0.0 |  |  |
|  | N137S, G275D | 0.35 / - |  | x |
| T276N |  |  |  |  |
|  | T276N | 0.0 / 0.0 | x |  |
| N276K |  |  |  |  |
|  | K62E, V144I, K156Q, E158K, V196A, N276K | 2.51 / 2.62 | x |  |
| T276I |  |  |  |  |
|  | T276I | 0.0 / - |  | x |
| T276A |  |  |  |  |
|  | R207K, L226P, I236T, T276A | 0.0 / - |  | x |
| I278S |  |  |  |  |
|  | S145N, Q189K, I217V, I278S | 0.57 / 2.42 | x |  |
| S278N |  |  |  |  |
|  | L226I, S278N | 0.1 / 0.0 | x |  |
|  | S278N | 0.0 / - |  | x |
|  | S278N | 0.49 / - |  | x |
| N278K |  |  |  |  |
|  | N278K | 0.74 / - |  | x |
| S278I |  |  |  |  |
|  | D188Y, N193K, S278I | - / 0.18 |  | x |
| I278V |  |  |  |  |
|  | I121T, P169S, I278V | 0.81 / - |  | x |
| S279Y |  |  |  |  |
|  | F94Y, E156K, S279Y | 0.74 / 0.51 |  | x |
| E280G |  |  |  |  |
|  | K62R, E158D, T192I, A198S, E280G | 0.0 / - |  | x |
| N285D |  |  |  |  |
|  | K135E, N145K, N285D | 1.62 / 1.55 |  | x |
| P289S |  |  |  |  |
|  | S219Y, P289S | 0.0 / - |  |  |
|  | P289S | 0.04 / - |  |  |
| D291G |  |  |  |  |
|  | D291G | 0.05 / - |  | x |
|  | D291G | 0.32 / - |  | x |
| D291N |  |  |  |  |
|  | N96S, R201K, K259Q, D291N | 0.0 / - |  | x |
|  | D291N | 0.83 / - |  | x |
| K292R |  |  |  |  |
|  | N171K, L226M, K264R, K292R | 0.66 / - |  | x |
| K299R |  |  |  |  |
|  | E82K, K83E, T131A, K299R | 0.81 / 0.29 | x |  |
| R299K |  |  |  |  |
|  | K92E, L226Q, R299K | 1.17 / 1.81 |  | x |
| A304P |  |  |  |  |
|  | T128A, A304P | 0.2 / - |  | x |
|  | S45N, F79L, A304P | 0.48 / - |  | x |
| K307R |  |  |  |  |
|  | N173K, I213V, N248T, K307R | 0.23 / 0.0 | x |  |
| V309G |  |  |  |  |
|  | S133N, S186I, V213R, V309G | 0.54 / - |  | x |
| V309I |  |  |  |  |
|  | V309I | 0.5 / 0.04 |  | x |
| K310R |  |  |  |  |
|  | G124D, G142E, D175E, K310R | 0.0 / 0.0 |  | x |
| N312K |  |  |  |  |
|  | V112I, N121T, N312K | 1.46 / 0.0 |  | x |
|  | G49S, K158R, V204I, Y233H, N312K | 0.0 / - |  | x |
| T313N |  |  |  |  |
|  | P103Q, T313N | 0.0 / - |  | x |
| V323I |  |  |  |  |
|  | V323I | 1.43 / 2.02 |  | x |
| E325G |  |  |  |  |
|  | E325G | 0.24 / - |  | x |
| E325D |  |  |  |  |
|  | D124E, K173R, R261H, E325D | 0.77 / 0.02 |  | x |
| K326R |  |  |  |  |
|  | D53N, K326R | 0.0 / - |  | x |
