# Supplementary material for: Inference of Genotype–Phenotype Relationships in the Antigenic Evolution of Human Influenza A (H3N2) Viruses
Source: PLoS Comput Biol. 2012 Apr 19;8(4):e1002492. doi: 10.1371/journal.pcbi.1002492 (PMC3330098; doi:10.1371/journal.pcbi.1002492)
Supplement: Table S3 — Positions with multiple changes in the phylogenetic tree and high antigenic weights (mean and median ≥1 antigenic unit, highlighted in bold). ‘Tip’ indicates leaf branches. (DOC) [file pcbi.1002492.s006.doc]

| Position | Up-weights (mean/median) | Down-weights (mean/median) | Trunk | Tip |
| --- | --- | --- | --- | --- |
| 112 | **1.14/1.13** | 0.30/0.18 | 0/4 | 4/4 |
| 137 | 0.21/0.15 | **1.27/1.05** | 3/6 | 3/6 |
| 144 | **1.21/1.39** | 0.95**/**0.41 | 4/9 | 4/9 |
| 155 | **1.52/1.77** | **2.29/3.16** | 3/3 | 0/3 |
| 156 | **1.28/1.44** | 0.79/0.00 | 1/3 | 2/3 |
| 189 | 0.45/0.57 | **1.95/2.42** | 3/3 | 0/3 |
| 208 | **1.24/1.73** | 0.59/0.59 | 0/3 | 2/3 |
