# Supplementary material for: Inference of Genotype–Phenotype Relationships in the Antigenic Evolution of Human Influenza A (H3N2) Viruses
Source: PLoS Comput Biol. 2012 Apr 19;8(4):e1002492. doi: 10.1371/journal.pcbi.1002492 (PMC3330098; doi:10.1371/journal.pcbi.1002492)
Supplement: Table S4 — Changes with multiple occurrences in the phylogenetic tree and high antigenic weights (mean and median ≥1 antigenic unit). ‘Tip’ indicates leaf branches. Down-weights are omitted, as all changes were identified using up-weights. (DOC) [file pcbi.1002492.s007.doc]

| Change | Up-weights (mean/median) | Trunk | Tip |
| --- | --- | --- | --- |
| K62E | 1.52/1.42 | 1/3 | 2/3 |
| V112I | 1.14/1.13 | 0/4 | 4/4 |
| N145K | 1.36/1.52 | 1/9 | 5/9 |
| L226Q | 1.16/1.07 | 1/8 | 6/8 |
| T248I | 1.01/1.48 | 0/3 | 3/3 |
