# Supplementary material for: Inference of Genotype–Phenotype Relationships in the Antigenic Evolution of Human Influenza A (H3N2) Viruses
Source: PLoS Comput Biol. 2012 Apr 19;8(4):e1002492. doi: 10.1371/journal.pcbi.1002492 (PMC3330098; doi:10.1371/journal.pcbi.1002492)
Supplement: Table S5 — Type-defining branches selected by different thresholds for average branch weights. Branches (1)–(9) were selected as type-defining branches at a threshold distance of 1.0 antigenic units. Branches (i)–(xii) reveal further subdivision of antigenic types at a threshold distance of 0.5 antigenic units. Asterisks mark branches whose sibling branch leads to a single isolate. Subscript 2 indicates that a branch is a direct successor of a type-defining branch (except for branch (i), which is a predecessor of the type-defining branch). Subscript sub indicates a subdivision of an antigenic type without a directly known reference strain. (DOC) [file pcbi.1002492.s008.doc]

| Threshold | No. | Type transition | Branch amino acid changes | Weights (up/down/avg) | Trunk |
| --- | --- | --- | --- | --- | --- |
|  | (1) | WU95–SY97 | K62E, V144I, K156Q, E158K, V196A, N276K | 2.5/2.6/2.6 | x |
| **2.0** | (2) | SY97–FU02 | L25I, R50G, H75Q, E83K, A131T, H155T, Q156H, S186G, V202I, W222R, G225D | 1.8/3.2/2.5 | x |
|  | (3) | BA79–SI87 | G124D, Y155H, K189R | 0.2/3.3/1.7 | x |
|  | (4) | VI75–TX77 | K50R, N137Y, G158E, M260I | 0.6/2.8/1.7 | x |
|  | (5) | HK68–EN72 | T122N, G144D, T155Y, R207K | 2.6/0.4/1.5 | x |
| **1.5** | (6) | EN72–VI75 | S145N, Q189K, I217V, I278S | 0.6/2.4/1.5 | x |
|  | (7) | BE92–WU95 | K135T, N145K, N262S | 1.5/1.1/1.3 | x |
|  | (8) | BE89–BE92 | I214T | 1.4/1.1/1.3 | x |
| **1.0** | (9) | SI87–BE89 | G135E, N145K | 2.0/0.0/1.0 |  |
|  | (i) | SI87–BE89_2_ |  | 1.0/0.9/0.9 | x |
|  | (ii) | BA79–CC85/LE86 | S159Y | 1.1/0.7/0.9 | x |
|  | (iii) | BE92_sub_ | N145K | 1.2/0.3/0.8 |  |
|  | (iv) | BE92–SH93 |  | 0.4/1.2/0.8 |  |
|  | (v) | BE92–JO94 | S47P, D124N, N216D, S219Y | 0.6/0.9/0.8 |  |
|  | (vi) | TX77–BA79 | N133S, P143S, G146S, K156E, T160K, Q197R, V217I | 1.4/0.0/0.7 | x |
|  | (vii) | BA79_sub_ | N2K, D144V | 1.4/0.0/0.7 | x |
|  | (viii) | BE92_sub_ | G135K | 0.2/1.1/0.7 | x |
|  | (ix) | FU02_2_* |  | 0.0/1.3/0.7 |  |
|  | (x) | SI87–GU89 | E82K, K83E, T131A, K299R | 0.8/0.3/0.6 | x |
|  | (xi) | EN72_2_* | L3F, N188D | 0.9/0.2/0.5 | x |
| **0.5** | (xii) | EN72–VI75_2_ | N53D, N137S, L164Q, F174S, N193D, R201K, I213V, I230V | 0.0/1.0/0.5 |  |
