# Supplementary material for: Inference of Genotype–Phenotype Relationships in the Antigenic Evolution of Human Influenza A (H3N2) Viruses
Source: PLoS Comput Biol. 2012 Apr 19;8(4):e1002492. doi: 10.1371/journal.pcbi.1002492 (PMC3330098; doi:10.1371/journal.pcbi.1002492)
Supplement: Text S1 — Influence of threshold distance on type-defining branches. (DOC) [file pcbi.1002492.s009.doc]

**Influence of threshold distance on type-defining branches**

The definition of antigenic types depends on the choice of the threshold distance. An average weight of 1.0 antigenic units as threshold distance resulted in robust antigenic types, differing at least by a two-fold dilution from the preceding antigenic type. Our method resolves antigenically relevant changes between successive antigenic types in several cases to several successive branches. Therefore, a higher threshold of 2.0 antigenic units for individual branches (a four-fold dilution), as suggested to distinguish antigenically diverse viral strains [1], does not allow distinction between different antigenic groups (only if the transition is not well resolved in the data and the antigenic impact of multiple changes is summarized on a single branch). Choosing a lower threshold distance of 0.5 antigenic units selected twelve further type-defining branches (**Table S5**, **Figure S3**). Although a direct interpretation of the threshold distance of 0.5 antigenic units is difficult, as it is not directly representative of dilution steps, five of these branches represent further subdivisions of antigenic types described before in literature. Among these branches is the branch leading to the TX77–BA79 transition that was also identified by antigenic cartography as a cluster transition [2]. Different from antigenic cartography, only a subset of the cluster-difference substitutions is assigned to this branch. The remaining changes account for intra TX77 changes and are assigned to the preceding branch.

Branch (i) is a predecessor of the SI87-BE89 type-defining branch. Although no change was assigned to this branch with a maximum likelihood character state reconstruction, with maximum parsimony ancestral character state reconstruction the change N193S was assigned to it. This branch also precedes the following BE92 type on the trunk, indicating that this precursor circulated undetected in the evolutionary reservoir during this time period and that N193S conferred a significant fitness effect. N193S is located in the receptor binding site [3] and, thus, probably represents an antigenically important substitution for SI87–BE89, which was not revealed by antigenic cartography. Similarly, branches (vii) and (viii) account for antigenic type differentiations and define unsampled antigenically distinct intermediates between two antigenic types. For both branches, several of the identified changes (G135K, D144V) occur at positions under positive selection [4], which further supports their antigenic relevance.

Other branches, such as (ii), define antigenic types that include viral isolates with sufficient antigenic dissimilarities to preceding viral strains to warrant vaccine updates. Here, branch (ii) refers to the antigenic type CC85/LE86, corresponding to two viral strains predominant from 1985 to 1987 [5-7]. With antigenic cartography, this type was placed within the BA79 cluster. Similarly, branch (iv) corresponds to the antigenic type SH93, branch (v) refers to the antigenic type JO94 and branch (x) refers to the antigenic type GU89. SH93 corresponds a viral strain predominant from 1993 to 1994 [8,9] and was placed within the BE92 cluster with antigenic cartography. JO94 corresponds to a viral strain predominant from 1994 to 1996 [10-13] and was placed within the BE92 cluster with antigenic cartography. Finally, GU89 corresponds to a viral strain recommended for use in the influenza virus vaccine in the 1990/91 northern hemisphere season [14] and was placed within the SI87 cluster with antigenic cartography.

The remaining branches account for intra-type antigenic differentiations with different interpretations. Branch (iii) indicates either an evolutionary volatile change with high antigenic impact or discrepancies between phylogenetic inference and antigenic phenotype (see discussion on isolates of the WU95 antigenic cluster placed in the BE92 cluster in the phylogenetic tree and on N145K in the main article). Branch (xii) is a successor of the VI75 type-defining branch and further distinguishes the VI75 antigenic type. The assigned changes are of antigenic relevance for distinguishing VI75 from previous and successive antigenic types but were assigned less antigenic impact than the changes assigned to the type-defining branch. Finally, branches (ix) and (xi) directly follow two type-defining branches and therefore further resolve these antigenic type transitions. Single isolates are separated by these branches from the remaining isolates of an antigenic type, indicating the presence of precursors with high antigenic distance to previous antigenic types. However, these branches show that further antigenic change resulted in the antigenic types that rose to predominance.

Although, some branches have no changes assigned to them, possible changes at the HA2 domain of the hemagglutinin or in the according neuraminidase may account for these antigenic variations. Furthermore, using a fixed tree topology that might be wrong, introduces further bias for fitting antigenic distances resulting in false assignments on branches without changes.

## References

1. Russell CA, Jones TC, Barr IG, Cox NJ, Garten RJ, et al. (2008) Influenza vaccine strain selection and recent studies on the global migration of seasonal influenza viruses. Vaccine 26: 31-34.

2. Smith DJ, Lapedes AS, de Jong JC, Bestebroer TM, Rimmelzwaan GF, et al. (2004) Mapping the antigenic and genetic evolution of influenza virus. Science 305: 371-376.

3. Wilson IA, Skehel JJ, Wiley DC (1981) Structure of the haemagglutinin membrane glycoprotein of influenza virus at 3 [angst] resolution. Nature 289: 366-373.

4. Bush R (1999) Positive selection on the h3 hemagglutinin gene of human influenza virus a. Mol Biol Evol 16: 1457-1465.

5. WHO (1986) Recommended composition of influenza virus vaccines for use in the 1986-1987 season. WHO Wkly Epidemiol Rec 61: 61-64.

6. WHO (1987) Recommended composition of influenza virus vaccines for use in the 1987-1988 season. WHO Wkly Epidemiol Rec 62: 54-56.

7. WHO (1987) Influenza. WHO Wkly Epidemiol Rec 62: 335.

8. WHO (1994) Recommended composition of influenza virus vaccines for use in the 1994-1995 season. WHO Wkly Epidemiol Rec 69: 53-56.

9. WHO (1994) Antigenic analysis of recent influenza virus isolates and influenza activity in the southern hemisphere. WHO Wkly Epidemiol Rec 69: 291.

10. WHO (1995) Influenza - antigenic activity of recent influenza virus isolates and influenza activity in the souther hemisphere. WHO Wkly Epidemiol Rec 70: 277.

11. WHO (1995) Recommended composition of influenza virus vaccines for use in the 1995-1996 season. WHO Wkly Epidemiol Rec 70: 53-56.

12. WHO (1996) Recommended composition of influenza virus vaccines for use in the 1996-1997 season. WHO Wkly Epidemiol Rec 71: 57-61.

13. WHO (1996) Antigenic analysis of recent influenza virus isolates and influenza activity in the southern hemisphere. WHO Wkly Epidemiol Rec 71: 292-293.

14. WHO (1990) Recommended composition of influenza virus vaccines for use in the 1990-1991 season. WHO Wkly Epidemiol Rec 65: 53-56.
