# Supplementary material for: Inference of Genotype–Phenotype Relationships in the Antigenic Evolution of Human Influenza A (H3N2) Viruses
Source: PLoS Comput Biol. 2012 Apr 19;8(4):e1002492. doi: 10.1371/journal.pcbi.1002492 (PMC3330098; doi:10.1371/journal.pcbi.1002492)

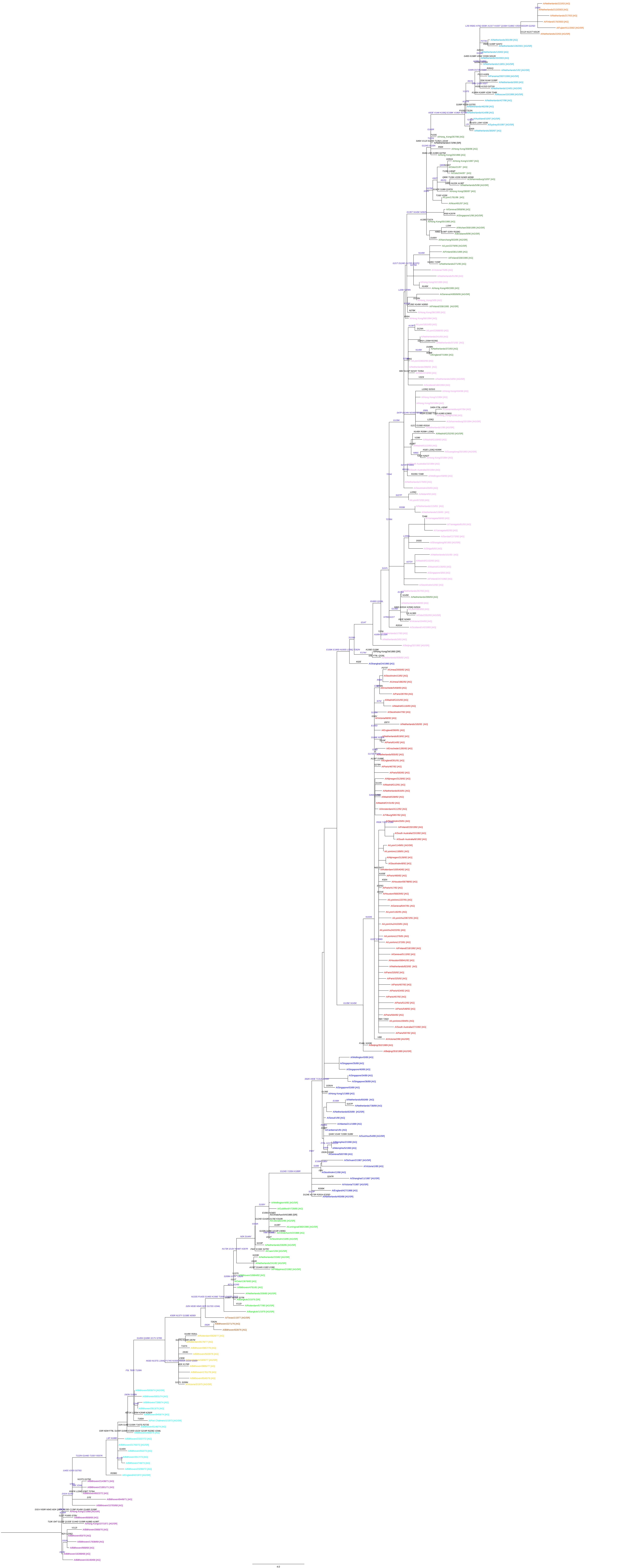

Supplement: Figure S1 — Antigenic tree with branch lengths representing antigenic distances (maximum of up- and down weights for each branch) inferred from a maximum likelihood tree of 258 hemagglutinin sequences of seasonal influenza A (H3N2) virus isolates and serological data. Isolates are color-coded by antigenic clusters according to Smith et al. (2004). Three isolates (A/Christchurch/4/85, A/Hong Kong/34/90 and A/Netherlands/172/96) are only present as antiserum and were not assigned a cluster label. Changes on terminal branches are colored in black, whereas changes on internal branches are colored in blue. (PDF) [file pcbi.1002492.s001.pdf]
