# Supplementary material for: Inference of Genotype–Phenotype Relationships in the Antigenic Evolution of Human Influenza A (H3N2) Viruses
Source: PLoS Comput Biol. 2012 Apr 19;8(4):e1002492. doi: 10.1371/journal.pcbi.1002492 (PMC3330098; doi:10.1371/journal.pcbi.1002492)

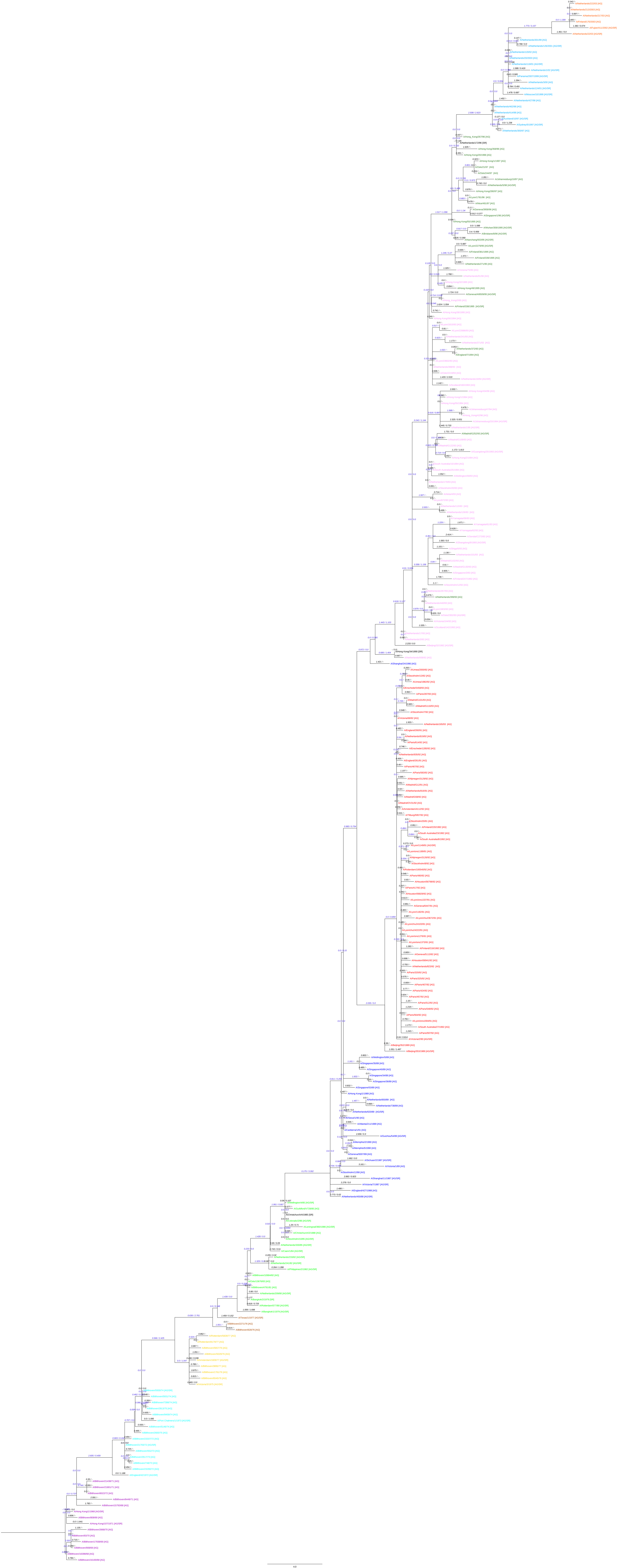

Supplement: Figure S2 — Antigenic tree with branch lengths representing antigenic distances (maximum of up- and down weights for each branch) inferred from a maximum likelihood tree of 258 hemagglutinin sequences of seasonal influenza A (H3N2) virus isolates and serological data. Isolates are color-coded by antigenic clusters according to Smith et al. (2004). Three isolates (A/Christchurch/4/85, A/Hong Kong/34/90 and A/Netherlands/172/96) are only present as antiserum and were not assigned a cluster label. Branch labels depict assigned weights (up/down). (PDF) [file pcbi.1002492.s002.pdf]
